# A Comprehensive and Structured Follow-Up for Persons With Multiple Sclerosis (CoreDISTparticipation) to Optimize Physical Functions, Health, and Employment: Protocol for a Prospective, Single-Blinded Randomized Controlled Trial and Health Economic Evaluation

**DOI:** 10.2196/74988

**Published:** 2025-10-08

**Authors:** Britt Normann, Marianne Sivertsen, Tonje B Braaten, Hans Olav Melberg, Hanne Kristin Fikke, Marianne Elvik, Ellen Christin Arntzen

**Affiliations:** 1 Department of Physiotherapy Nordland Hospital Trust Bodø Norway; 2 Faculty of Nursing and Health Science Nord University Bodø Norway; 3 Department of Community Medicine UiT The Arctic University of Norway Tromsø Norway; 4 The Norwegian MS Association Bodø Norway

**Keywords:** multiple sclerosis, rehabilitation, physical functioning, physical activity, employment

## Abstract

**Background:**

Multiple sclerosis (MS) is a chronic neurological disease of the central nervous system, primarily affecting young adults. Common challenges in MS include fatigue, physical impairments, and cognitive impairments, associated with low levels of physical activity, unemployment, reduced health-related quality of life (HRQoL), and substantial personal and societal costs. Many leave the workforce or reduce hours even when disability is low and despite a desire to increase work hours if the job is adjusted to their needs. Existing services aiming to optimize physical functions and work participation only initiate retrospectively, and there is a lack of knowledge regarding the possible effect of more proactive services.

**Objective:**

The objective of this study is to investigate the effects of a comprehensive multidisciplinary intervention, CoreDISTparticipation, delivered across health care levels (hospitals and municipalities) and sectors (health and employment/welfare), on barriers to work, physical activity, and physical functions; fatigue; and HRQoL for employed people with multiple sclerosis (pwMS) and to perform a health economic evaluation.

**Methods:**

This prospective, single-blinded randomized controlled trial (RCT) will include 115 pwMS with Expanded Disability Status Scale (EDSS) scores of 0-4 randomly allocated to either a CoreDISTparticipation intervention group or usual care (control group). The CoreDISTparticipation intervention includes (1) information videos, hospital outpatient physiotherapist assessments, and meetings with employment consultants; (2) group-based physiotherapy in municipalities for 60 minutes over 6 weeks, one indoor CoreDIST balance session, one outdoor CoreDIST balance and high-intensity interval session, and tailored work follow-up; and (3) 6 weeks of digitally supported independent training, twice weekly. Assessments will be conducted at baseline, week 9, and week 16. Primary outcomes include Multiple Sclerosis Work Difficulties Questionnaire-23 – Norwegian version (MSWDQ-23NV) and ActiGraph wGT3x-BT monitor scores. Secondary outcomes include Trunk Impairment Scale – modified Norwegian Version (TIS-modNV), Mini Balance Evaluation Systems Test (MiniBESTest), AccuGait Optimized force platform, 6-minute walk test (6MWT), Multiple Sclerosis Walking Scale‐12, Multiple Sclerosis Impact Scale-29 – Norwegian version, EQ-5D-5L, and Fatigue Severity Scale – Norwegian version scores. The study will identify effects of CoreDISTparticipation versus usual care on work barriers, physical activity, balance, walking, fatigue, and quality of life, along with a health economic evaluation. Descriptive statistics and repeated measures mixed models will be performed using IBM SPSS version29.

**Results:**

We completed the enrolment phase and enrolled and randomized 115 participants in two phases by August 1, 2024. The 15-week retests were completed in December 2024, and data collection is estimated to be completed by September 2025. Results are expected to be published in the first quarter of 2026.

**Conclusions:**

CoreDISTparticipation is an innovative approach proactively addressing physical functions, physical activity, and work participation. If effective, it can offer a low-cost approach that potentially may enhance the quality of life and workforce sustainability and reduce societal costs.

**Trial Registration:**

ClinicalTrials.gov NCT06110468; https://www.clinicaltrials.gov/study/NCT06110468

**International Registered Report Identifier (IRRID):**

DERR1-10.2196/74988

## Introduction

### Background

Multiple sclerosis (MS) is a chronic neurological disease of the central nervous system that primarily affects young adults [1]. Approximately 2.9 million people worldwide have MS [2], and Norway is a high-risk area [2] with a prevalence rate of 248/100,000 and an incidence of 10/100,000 [3,4]. Common challenges in MS include fatigue, physical impairments, and cognitive impairments [3], which are associated with low levels of physical activity, unemployment, reduced health-related quality of life (HRQoL), and substantial personal and societal costs [5-7]. In 2022, the socioeconomic costs in Norway related to people with multiple sclerosis (pwMS) amounted to 12 million Norwegian Kroner (NOK), or ~US $120,520, with about one half attributed to loss of productivity and around NOK 900,000 (US $90,390) per person [4]. Given the median survival rate of 48 years postdiagnosis [3], around 30 years of their working life could potentially remain after receiving the diagnosis.

In Norway, most pwMS experience mild-to-moderate disability, as measured with the Expanded Disability Status Scale (EDSS) [3]. Despite low disability, the disease course fluctuates with the accumulation of minor impairments, such as fatigue [7], impaired balance and walking [8,9], and cognitive problems [10]. Physiotherapy and physical activity interventions can reduce fatigue [8,9]; improve balance, walking [10-13], and HRQoL [9,14,15]; and enhance neuromuscular and physical functioning in pwMS [16]. Optimizing physical functions and physical activity when disability is low, and neuroplasticity is optimal [17], can be valuable for maintaining physical activities and work, as less fatigue, mobility-related symptoms, and cognitive disturbances are associated with current employment [18-23].

It is concerning that 40%-70% of pwMS exit the workforce within a few years postdiagnosis [7,24-26], even with minor disability, as measured with the EDSS [4,5,18,19,27]. Nonemployment rates tend to increase with age [28], impacting on identity, self-esteem, self-efficacy, and HRQoL [14,29]. Barriers to work include demographic-, disease-, and workplace-related factors [15,30]. A higher age at onset of MS, a longer disease duration, lower education, fatigue, heavy physical work, depressive symptoms, and high levels of disability are associated with unemployment [18,19,27]. In Norway, 54%-70% of pwMS are outside the workforce [6,18]. Interestingly, our recent pilot feasibility randomized controlled trial (RCT) [31], with 28 participants with an average EDSS score of 1.8 (SD 1), identified a prevalent desire to increase work hours if the jobs were adjusted to their needs. The mean desired employment rate was 72.7% (SD 26.3) for the intervention group and 83.8% (SD 22.6) for the usual care group in contrast to their current mean employment rate of 46.3% (SD 35.6) and 65.4% (SD 39.3) [31], respectively. These findings highlight the potential for pwMS to remain in the workforce for longer with adequate support.

The Norwegian Labour and Welfare Administration (NAV) is responsible for all employment and welfare services. NAV is a result of the merging of three previous public institutions: Employment Services, Social Insurance Administration, and Municipal Social Services. Support from NAV is initiated 26 weeks after a person is prescribed sick leave, with the employer being responsible for initial work adaptation. Communication between pwMS and their managers is essential for sustained employment [17,21]. Employers, however, often lack relevant information about the disease and adequate work adaptations [32], and NAV consultants report challenges, such as limited time, lack of information, and difficulties in understanding people with chronic diseases [33]. At 26 weeks, NAV consultants offer a dialogue meeting to discuss return-to-work options [34], potentially delaying adequate actions for 6 months. This practice, which initiates actions *retrospectively* and is carried out by actors with limited competence regarding MS, needs improvement.

To address these issues, we have developed a comprehensive intervention entitled *CoreDISTparticipation*. This multidisciplinary follow-up is delivered across health care levels (hospitals and municipalities) and sectors (health and employment/welfare). It builds on the GroupCoreDIST exercise intervention [35], which addresses prerequisites for balance, such as dynamic trunk control and coordination within and between proximal (core) and distal bodily areas, delivered in small groups. These exercises emphasize high dose, dual task (D); individualization and new insights (I); somatosensory function optimization (S); and task-oriented training (T). GroupCoreDIST has proven effective for trunk control, balance, and walking in the short and the long term [12,13]; is a valuable tool for physiotherapists [36-38]; and enhances pwMS engagement in physical activity [39]. In addition, CoreDISTparticipation incorporates structured support from NAV consultants and employers, as well as digital support, and emphasizes outdoor physical activity. This innovative intervention has been investigated through a feasibility pilot RCT [31], a pretest-posttest feasibility study [32], and interviews [40]. Following minor adjustments, it is ready for a large-scale RCT. Currently, there is a scarcity of evidence regarding proactive comprehensive interventions addressing function, health, and work in pwMS. The trial presented in this protocol will investigate the effectiveness and cost-effectiveness of the new intervention.

### Objectives

We pose the overall research question: What are the effects of CoreDISTparticipation on physical functions, health, and employment compared to usual care for pwMS? Subquestions organized in two work packages (WPs 1 and 2) are outlined in Table 1.

Our hypothesis is that CoreDISTparticipation is superior to usual care in terms of addressing barriers to and status of work and improving physical activity, physical functions, fatigue, and HRQoL. It is, however, important to note that the initial phase of the intervention may entail an increase in short-term sick leave costs due to the intensive program that may need to be conducted during working hours.

**Table 1:** Research questions for work packages WP^a^1 and WP2.

WP	Research questions
WP1	What are the short- and long-term effects of the CoreDISTparticipation program compared to usual care regarding barriers to work, physical activity, balance, walking, fatigue, and HRQoL^b^ in individuals with minor-to-moderate disability due to MS^c^? What are the distributions of and relationships between barriers to work and physical activity, balance, walking, fatigue, HRQoL, and employment status in individuals with minor-to-moderate disability due to MS?
WP2	What is the utility of the CoreDISTparticipation program compared to usual care expressed in the unit of QALYs^d^ in individuals with MS? What is the utility of the CoreDISTparticipation program compared to usual care according to long-term employment status and work-related costs in individuals with MS?

^a^WP: work package.

^b^HRQoL: health-related quality of life.

^c^MS: multiple sclerosis.

^d^QALY: quality-adjusted life-year.

## Methods

### Trial Design

This study consists of two WPs. WP1 is a parallel, two-arm prospective, single-blinded, multicenter RCT with an allocation ratio of 1:1 to investigate whether CoreDISTparticipation is superior to usual care in terms of reducing barriers to work and improving physical activity levels, physical functions, and HRQoL. The study is registered at ClinicalTrials.gov (NCT06110468). WP2 aims to determine the utility of the CoreDISTparticipation intervention in terms of HRQoL and quality-adjusted life-years (QALYs), employment status, and work-related costs when compared to usual care. The trial protocol is reported in line with the SPIRIT (Standard Protocol Items: Recommendations for Interventional Trials) checklist (Multimedia Appendix 1) and the CONSORT statements for reporting RCTs.

### Study Setting

The trial will be conducted across levels of health care and institutions, specifically in hospitals and municipalities, in outpatient clinics, in an outdoor setting, and in the participants’ home. All testing and initial physiotherapy assessments for the intervention group will be undertaken in a hospital setting. The physiotherapy intervention involves a combination of indoor and outdoor training and will take place in municipality physiotherapy outpatient clinics and appropriate outdoor venues. Work-related meetings will preferably be digital but can be held in the NAV consultants’ offices or at the participants’ place of work.

### Eligibility Criteria

Eligible participants must be diagnosed with MS according to the McDonald criteria [41], have an EDSS score of ≤4, be between 18 and 67 years of age, be employed (10%-100%; may include various degrees of sick leave, disability pension, or Work Assessment Allowance [WAA]), and be living in 1 of the 22 participating municipalities within the regions served by the participating outpatient clinics. Exclusion criteria are pregnancy at enrollment, exacerbation of symptoms (ie, relapse) within 2 weeks prior to enrollment, or other serious acute conditions.

### The CoreDISTparticipation Intervention

#### Choice of Comparators

We have chosen usual care to be the most relevant comparator, even though it is unlikely to be dose-matched, as it represents current practice. These participants will continue with their regular routines and will be encouraged to obtain the national and MS-specific recommendation of 150-300 minutes of moderate physical activity or 75 minutes of high-intensity physical activity per week or a combination of these, stay employed, continue medical treatment, and seek any health care required, including physiotherapy.

#### The Intervention

The intervention (Table 2) will consist of information videos on the importance of physical activity and possibilities for work adaptations, individual physiotherapy assessments and indoor and outdoor training in groups, meetings with a NAV consultant to assess the need for work adaptations or support and subsequent follow-up with the participants’ leader/manager, digitally supported self-administered home exercises, and a podcast on physical activity.

**Table 2:** Overview of the CoreDISTparticipation intervention.

Phase and weeks			Description
**Phase 1**			
	Week 1	The pwMS^a^ and their employers watch three short videos. One addresses information regarding MS^b^ and another on how NAV^c^ can provide support in terms of work adaptations. The third video contains information from both physiotherapists and a user representative regarding the importance of training physical functions and regular physical activity. A digital work-promoting meeting between the pwMS and a trained NAV consultant following a structured guide (60 minutes): This aims to identify the individual’s needs for follow-up regarding work adaptations. The meeting will determine whether the pwMS independently should discuss the work situation with their employer or whether a NAV consultant, a general practitioner, or other professional is needed in the follow-up. A clinical assessment with a trained MS outpatient physiotherapist (60 minutes): This explores the individuals’ movement challenges and explores possibilities for optimalization. Information will be sent digitally to the municipal physiotherapist.	
	Weeks 2-9	A clinical assessment with the municipal physiotherapist (90 minutes): This should build on the documentation from the MS outpatient physiotherapist. Focus is on exploring possibilities for change related to their movement problem to enable them to master the principles of the CoreDIST exercises, especially related to core activation in coordination with distal movement, and identify goals for physical functions, physical activity, and work. The goals will be written and evaluated after the 6-week period. The CoreDIST videos [42] and outdoor voiced programs will be introduced, and the pwMS will discuss what physical activity they prefer. Conclusions regarding goal setting will be sent digitally to the general practitioner. CoreDIST sessions two times per week for 6 weeks (1×60 minutes indoor training in groups of 1-5 participants and 1×60 minutes outdoor training in groups of 1-10 participants). The indoor exercises will focus on optimalization of prerequisites for balance, such as improving somatosensory activation, muscle length, activation of trunk muscles in coordination with distal movement, larger muscle groups and postural control challenges in standing. The outdoor exercises will focus on challenging postural control in standing and moving, as well as physical capacity in high-intensity intervals (4×4minutes) of walking or running or both. A pulse monitor will be worn, and intensity will be individualized. Self-administered training 1×30 minutes per week, eHealth-supported CoreDIST videos, voiced programs, and a podcast are available. Self-administered training will be registered on a weekly basis through SMS reminders. A (digital) follow-up meeting regarding work between the pwMS and employers or arranged by NAV with the participants, employers, and professionals relevant for the patients. Week 9: A physical meeting with the municipal physiotherapist (30 minutes) to evaluate goals and new goal setting for weeks 10-24 regarding physical functions and physical activity. The municipal physiotherapist sends information regarding goals to the general practitioner.	
**Phase 2**			
	Weeks 10-15	Self-administered, digitally supported home training: CoreDIST videos, 3×10 minutes/week Physical activity and training of own choice, 2×30 minutes/week; for instance, using outdoor training with provided voiced programs. The training should include elements of high intensity and balance. SMS reminder will be sent on a weekly basis from the Nordland Hospital Trust. Participants will register the number of training and physical activity sessions by responding to a digital questionnaire on a weekly basis. Week 15: Evaluation of work-related goals (30 minutes): The pwMS, employers, and potentially other professionals involved in goal setting/follow-up will evaluate physical activity; the meeting is in consultation with the municipal physiotherapist (30 minutes). The information will be sent to the general practitioner.	

^a^pwMS: people with multiple sclerosis.

^b^MS: multiple sclerosis.

^c^NAV: Norwegian Labour and Welfare Administration.

#### CoreDIST Exercises

##### Indoor Training

**Figure 1:**
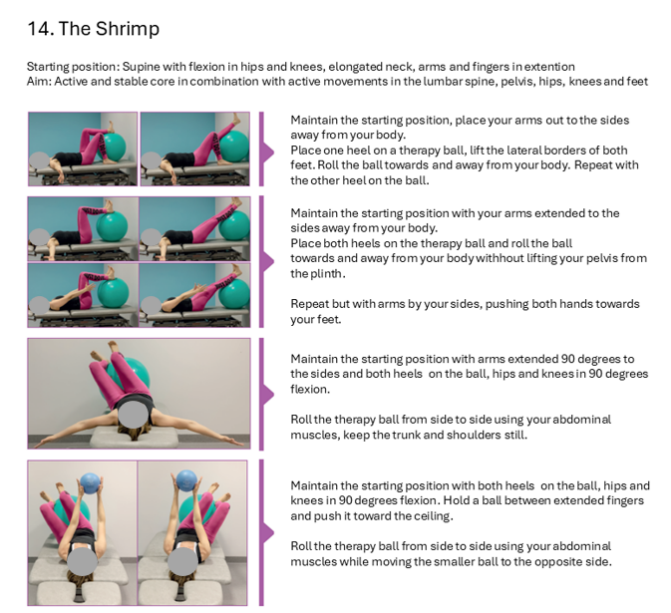
Example of exercise description.

The intervention includes 38 standardized exercises, which will be performed in various positions (Multimedia Appendix 2). These exercises are organized in eight “blocks” according to their objectives, and all are individually named and described (Multimedia Appendix 3). Each exercise includes three to five levels of difficulty to allow for individual tailoring (Figure 1). In the group sessions, all participants perform the same exercise simultaneously, but the level of difficulty may be individually adjusted. The intervention emphasizes performance of exercises with optimal alignment in various body parts to enhance recovery as opposed to compensatory strategies [43]. Dynamical proximal stability is enhanced in most exercises by keeping the back, hips, or stomach in dynamic contact with a therapeutic ball (smaller balls may also be used). Trunk muscles are addressed in all exercises, for instance, by keeping the ball still against a wall while moving the arms or legs or by moving the ball while keeping the hands or feet stable. Sensory stimulation of hands and feet to update body schemas is facilitated by using a spiky ball or roller [43,44]. Dual tasks are integrated as variations, for example, by singing, using rhymes, or calculating while exercising. Proprioception and vestibular functions are enhanced by performing exercises with closed eyes. Interacting ball activities, such as bouncing, jumping, and throwing the ball to each other, are included to increase postural control challenge and enhance group dynamics.

##### Outdoor Training

The CoreDIST exercises, along with the principles of 4×4-minute intervals form the basis for the outdoor sessions (Multimedia Appendix 2). There is a strong emphasis on high intensity [45] through running or brisk walking or performing exercises when standing with resistance or at a faster pace.

#### Meeting With the NAV Consultant

A written themed guide for the meetings between participants and NAV consultants have been developed in collaboration with NAV and user representatives (Multimedia Appendix 4).

#### Participant Timeline

**Figure 2:**
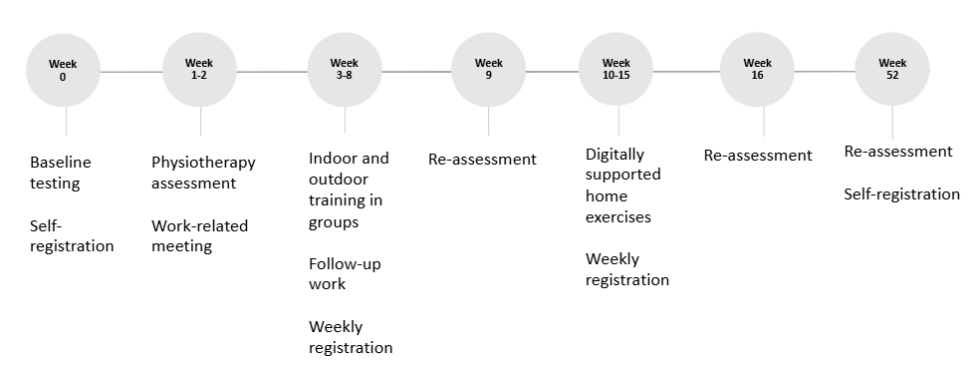
Participant timeline. NAV: Norwegian Labour and Welfare Administration.

The timeline for participants in the intervention group is outlined in Figure 2. Participants in usual care (control group) will undertake a baseline assessment and retests, along with registrations in weeks 9, 16, and 52.

#### Criteria for Discontinuing or Modifying the Allocated Intervention

All participants may withdraw from the study without providing a reason, and this will not affect their access to care. Participants will not be able to change their group allocation. With the participants’ consent, all data collected up to withdrawal will be included in the analysis. Relapses or medication changes will be registered but are not reasons for exclusion, provided the participants are willing and able to continue.

#### Strategies to Improve Adherence to the Intervention

Several strategies have been developed to improve and monitor adherence. Members of the research team will be available to answer questions or provide support to the participants and physiotherapists during working hours throughout the project period and may be contacted over the phone or via email. NAV consultants and physiotherapists will contact the participants over the phone to set up appointments. To facilitate adherence during the 6-week period of self-administered training, weekly reminders and activity suggestions will be distributed. The participants will perform weekly registrations of the number of sessions and intensity in training. They will also be provided with access to a digital exercise bank. The participants in the intervention group will be provided with fitness watches for the purpose of pulse monitoring and logging during sessions, as well as for setting individual physical activity goals, such as a daily step count.

#### Strategies to Improve the Fidelity of the Study

All participating physiotherapists will participate in a 3-day training program on the CoreDISTparticipation intervention. The training will focus on the theoretical basis and practical execution of the intervention. The physiotherapists will be provided with a booklet containing images and descriptions of all exercises (see Figure 1), including possibilities for adjusting the level of difficulty in each exercise. They will register all exercises used during each training session. The NAV consultants involved in the delivery of the intervention will participate in an online training course providing basic knowledge of MS. The participants will log their average heart rate and time spent in different pulse zones during the physiotherapy sessions on forms kept by their physiotherapist. This will aid the physiotherapists in achieving sufficient intensity in the sessions.

The participants will be enrolled into the study in two groups, where one group will finish the intervention before the other group starts. Evaluation meetings with the testers, physiotherapists, and NAV consultants will be held before the enrollment of group 2 to address any issues they encounter with group 1.

### Relevant Concomitant Care Permitted or Prohibited During the Trial

No restrictions will be imposed on the participants in either group during the study period. The participants will register treatments related to their MS, including rehabilitation treatments.

#### Provisions for Posttrial Care

All participants may continue to receive routine care from the MS outpatient clinic and multidisciplinary care, as needed. No other arrangements will be made for provision of posttrial care. All participants will be covered by the Norwegian Patient Injury law, providing entitlement to apply for compensation from the Norwegian System of Patient Injury Compensation in the case of any harm while participating in the study.

### Outcomes

#### Work Package 1 (RCT)

An overview of the selected valid and reliable outcome measures that will be used is outlined in Table 3. The primary outcomes will be the Multiple Sclerosis Work Difficulties Questionnaire-23 – Norwegian version (MSWDQ-23NV) scores and physical activity levels measured by ActiGraph wGT3x-BT monitors. We have chosen these primary outcomes as they cover the main parts of the intervention. The secondary outcomes cover factors associated with reduced employment and HRQoL, along with specific insights into gait capacity, balance, and trunk control.

All outcome measurements will be conducted at baseline and weeks 9, 16, and 52 to examine short- and long-term within- and between-group effects, as outlined in Table 4.

Two trained physiotherapists (research assistants), blinded to group allocation and not involved in the delivery of the intervention, will perform the assessments in the local hospitals near the municipalities where the participants live, and distribute the activity monitors. Questionnaires will be administered digitally through the Research Electronic Data Capture (REDCap) tool. The participants will provide information regarding the type and duration of MS, as well as their age, gender, smoking status, weight, social status, education, current work, employment status, well-being, and motivation for work and physical activity. The patients will also self-report MS attacks, physical activity (type, dose, frequency, intensity), medications, physiotherapy, and work-related meetings.

**Table 3:** Outline of the primary and secondary outcome measures for WP^a^1.

Outcome and measures			Description
**Primary outcomes**			
	MSWDQ-23NV^b^	This measures how frequently individuals with MS^c^ perceive psychological/cognitive (11 items), physical (8 items), and external (4 items) barriers related to work, scored on a 5-point scale (0=best) [46].	
	Levels of physical activity measured using ActiGraph wGT3x-BT monitors	The monitors will be worn on a belt around the waist for 7 consecutive days. The average daily percentage of time in mild, moderate, and intensive physical activity and sedative time will be calculated [47].	
**Secondary outcomes**			
	6MWT^d^	This measures the distance in meters walked within 6 minutes [48].	
	ActiGraph wGT3x-BT monitor	This counts the average number of steps taken per day.	
	EQ-5D-5L + complementary questions	This is the self-perceived HRQoL^e^ in five domains and a Visual Analogue Scale (score 0-100) on overall health [49]. Complementary questions will be asked on sleep, well-being, emotions, and social relations (advocated by the Norwegian Health Institute). A higher score indicates better self-perceived health.	
	Fatigue Severity Scale - Norwegian version	This is a self-report, 9-item scale that measures the physical, social, and cognitive effects of fatigue (score 1-7). A higher score indicates higher levels of fatigue [50].	
	Multiple Sclerosis Walking Scale-12	This 12-item scale measures the self-reported perception of walking, scored on a 5-point scale. A higher score indicates a higher impact of MS on the individual’s walking ability [51].	
	MiniBESTest^f^	This test involves pro-and reactive balance, dual tasks, and involving sit-to-stand, standing, and walking tests. There are 14 items scored on a 3-point scale. A higher score indicates better performance [52].	
	TIS-modNV^g^	Trunk control in sitting: This 6 item scale is scored on a 2- or 3-point scale (sum range 0-16 points). A higher score indicates better trunk control [53].	
	AccuGait Optimized force platform	This measures postural control in standing and one-leg standing: Balance in a quiet stance is expressed through the COP^h^ migration and amplitude of postural sway [54].	
	Multiple Sclerosis Impact Scale-29 – Norwegian version	This measures the self-perceived physical (13 items) and psychological (9 items) impact on HRQoL using a 5-point scale. A higher score indicates an increased impact of MS on the individual’s day-to-day life [55].	
	Patient Global Impression of Change (PGIC) – physical activity and balance	This global index is used to rate the response of a condition to a therapy (transition scale). Higher scores indicate self-perceived improvement [56].	

^a^WP: work package.

^b^MSWDQ-23NV: Multiple Sclerosis Work Difficulties Questionnaire-23 – Norwegian version.

^c^MS: multiple sclerosis.

^d^6MWT: 6-minute walk test.

^e^HRQoL: health-related quality of life.

^f^MiniBESTest: Mini Balance Evaluation Systems Test.

^g^TIS-modNV: Trunk Impairment Scale – modified Norwegian version.

^h^COP: center of pressure.

**Table 4:** Overview of registrations and assessments.

Registration and assessment	Baseline	Weekly	Week 9	Weekly	Week 16	Monthly	Week 52
Standardized outcome measures	X^a^	—^b^	X	—	X	—	X
Physiotherapy and work-related meetings (baseline and week 52: report the last 3 months)	X	—	X	—	X	—	X
Current work, employment status, well-being	X	—	X	—	X	—	X
Self-reports: MS^c^ attacks, physical activity, medications physiotherapy, general practitioner, NAV^d^ consultations	X	X	X	X	X	X	X

^a^X: applicable.

^b^Not applicable.

^c^MS: multiple sclerosis.

^d^NAV: Norwegian Labour and Welfare Administration.

#### Work Package 2 (Utility Study)

The outcomes for WP2 will be the net utility gain, employment status, and cost utility (Table 5). Data related to participation in work will be obtained cross-sectionally at baseline in WP1 and after week 52 through self-registration and linkage to the NAV register. The participants will provide information about the type and number of follow-ups and treatments from the physiotherapist, the general practitioner, the neurologist, and the NAV consultant in digital questionnaires distributed from REDCap. From the NAV registry, data will be obtained for the following variables: work status, sick leave, WAA and disability benefits, family-related leave (ie, maternity leave), pension, social aid, and unemployment benefits.

**Table 5:** Outline of outcomes for WP^a^2.

Outcome	Description
Net utility gain: QALYs^b^	QALYs will be calculated for both groups using EQ-5D-5L scores from WP1.
Employment status	The status for and changes in employment will be calculated based on self-reports and registry data from NAV^c^. To enable calculation of long-term effects, we will gather data on the aforementioned variables 1 year after the intervention.
Cost utility	Data on treatment costs will be obtained from the tariffs and weights used in municipal and specialist health care.

^a^WP: work package.

^b^QALY: quality-adjusted life-year.

^c^NAV: Norwegian Labour and Welfare Administration.

### Sample Size

We calculated the sample size needed to detect a between-group difference, as observed in our pilot study [31], based on the MSWDQ-23NV scores. The mean score change from baseline to posttest was 5.9 for the intervention group and 2.3 for the control group [31]. Considering the observed SDs and covariances and calculating with a power of 0.8 and α=.05, the RCT will need to include 45 individuals in each group. Assuming a dropout rate of 20%, we decided to recruit 115 people with mild-to-moderate disability due to MS into the study.

### Recruitment

Participants were recruited from the neurological outpatient clinics in three hospitals in Norway. Based on the prevalence of pwMS and the prospects of being able to recruit sufficient participants to form groups, only municipalities with >4500 inhabitants (n=19) or smaller municipalities (n=3) near an outpatient clinic in a neighboring municipality were invited to participate. Based on patient lists from the participating outpatient clinics, all individuals in the selected municipalities who met the EDSS score criterium received an invitation to participate.

### Assignment of Interventions: Allocation

#### Sequence Generation

Digital randomization to CoreDISTparticipation (intervention group) or usual care (control group) in a 1:1 ratio using block sizes of 4 and 6 with a random variation between the two block sizes was performed. Randomization was stratified based on the participants’ region of residence (north or south) to optimize the establishment of exercise groups and avoid geographical bias. Randomization was performed using REDCap hosted by the Clinical Research Department, University Hospital North Norway, a secure web-based app designed to support data capture for research studies, providing (1) an intuitive interface for validated data entry, (2) audit trails for tracking data manipulation and export procedures, (3) automated export procedures for seamless data downloads to common statistical packages, and (4) procedures for importing data from external sources [57].

#### Concealment Mechanism

The allocation sequence was generated outside REDCap and uploaded to the REDCap database prior to enrollment of participants and was not visible to the members of the research group performing the randomization.

#### Implementation

The allocation sequence was generated by an administrative member of staff at the Research Department at the Northern Norway Regional Health Authority. This member of staff is otherwise not connected to the project and will not reveal the allocation sequence to any member of the research team. A member of the research group not involved in assessment or treatment of the participants or in the generation of the allocation sequence enrolled participants into the REDCap database upon screening them based on the inclusion and exclusion criteria and whether they provided informed consent. Following baseline measurements, digital randomization to the intervention or the control group was performed.

### Assignment of Interventions: Blinding

In line with many rehabilitation studies, this will be a single-blinded study. The outcome assessors will be blinded to group allocation. All participants have been instructed not to reveal their group allocation to the outcome assessor. Blinding of participants will not be possible as there is no control group intervention. No procedures for unblinding are planned as this is a single-blinded study.

### Data Collection and Management

#### Plans for Assessment and Collection of Outcomes

Demographic data on age, gender, height, weight, social status, education, medical history (including the type of MS, years since diagnosis, EDSS score and MS-related medications), work status, and physical activity will be collected from each participant digitally through Nettskjema’s online forms. All questionnaires will be distributed digitally from the REDCap database, and the participants will be notified via email. The clinical tests will be performed at the patients’ local or regional hospital. Two assessors will be trained in all tests to be performed, and training will include calibration of the two assessors. Scores from the clinical tests will be noted on a paper form prior to entry into the REDCap database.

#### Plans to Promote Participant Retention and Complete Follow-Up

All visits to the hospitals, municipality physiotherapists, and NAV consultants are free of charge, and travel costs will be reimbursed. Trial meetings with the participating physiotherapists and NAV consultants will be held before, during, and after the intervention to address questions. All questionnaires will be distributed digitally, thus allowing the participants to answer them in their own pace and to allow for breaks.

#### Data Management

Digital consent forms and demographic data collected via online forms will be stored securely at the project database at the Tjenester for Sensitive Data (Services for Sensitive Data; TSD). Data for primary and secondary outcome measures will be collected and managed using REDCap. All patient-reported outcome measures (PROMs) will be distributed digitally from REDCap, and responses will automatically be entered into the REDCap database. Point scores for the Trunk Impairment Scale – modified Norwegian Version (TIS-modNV), the Mini Balance Evaluation Systems Test (MiniBESTest), and the 6-minute walk test (6MWT) will be entered manually into the REDCap database. Paper score sheets for clinical tests will be stored in a locked cabinet. Deidentified data stored at the TSD and in the REDCap database will be exported securely into a secure server at the Nordland Hospital Trust prior to statistical analysis. All data will be coded with the participants’ study IDs. A list of participants containing both names and study IDs will be sent to NAV, and NAV will return the registry data for all participants with study IDs but without names. All files will be sent securely as password-encrypted files via email; passwords will be sent separately via text messages. The files will be stored at the Nordland Hospital Trust’s secure server for research data. The data management plan is stored in the Trial Master File.

#### Confidentiality

Lists containing all patients with a diagnosis of MS will be collected from the MS outpatient clinics. These lists will be sent via email as password-encrypted files, and the passwords will be sent separately as text messages. Members of the study group will screen the lists against the inclusion and exclusion criteria through access to the hospital journals. Each participant will be assigned a study ID, and only the study ID will be entered into the study database. A key connecting the study ID to the participant’s name will be stored separately in the secure study database and will only be accessible to two members of the study group.

#### Analysis

First, the study aims to generate new knowledge regarding the short- and long-term effects of the CoreDISTparticipation intervention compared to usual care on barriers to work, physical activity, balance, walking, fatigue, and HRQoL in employed individuals with minor-to-moderate disability due to MS. As the intervention is complex and comprises several elements, the study will investigate the overall effects. The variables examined are well-known barriers to work and quality of life in this population [30]. Understanding the relationships between these and the intervention’s potential effects will provide valuable knowledge for further development of follow-up care.

Second, this study will provide objective information about health economic factors related to pwMS with low disability. This includes the utility of the CoreDISTparticipation program compared to usual care, expressed in QALYs, long-term employment status, and work-related costs. Given the high work-related costs associated with pwMS [4,7], this study will offer essential insights to further develop follow-up care and potentially enhance workforce sustainability for this group. Thus, the study addresses the part of the follow-up that represents the highest societal costs for pwMS in Norway [4].

#### Data Analysis

Raw data from the ActiGraph wGT3x-BT monitors will be converted into categories of minutes of sedative time, minutes of light and moderate-to-vigorous physical activity, and the number of steps per day using ActiLife software. The raw platform data will be filtered using a fourth-order Butterworth filter at 10 Hz. Center of pressure (COP) data will be calculated using BalanceClinic software and exported to MatLab, where the root mean square (values) representing postural sway amplitude will be calculated. Multiple imputations of missing data in PROMs will be applied, when appropriate. EQ-5D-5L answers will be coded into digits according to the recommendations in the instrument handbook. Value sets representative for the Norwegian population will be used in the calculation of a single summary index value.

#### Statistical Analysis for Primary and Secondary Outcomes

##### Work Package 1

Intention-to-treat analysis using descriptive statistics and, when appropriate, mixed models will be used to determine possible between-group differences over repeated measurements. Descriptive statistics including graphical methods, nonparametric tests, correlation, and regression analyses will be performed.

##### Work Package 2

The EQ-5D-5L (from WP1) is the primary outcome measure used in this analysis, which will be supplemented with generic complementary questions. Responses from the EQ-5D-5L will be used to examine the overall net utility gain from the CoreDISTparticipation program compared to usual care. Cost utility analysis is a form of economic evaluation that uses quality-of-life measurements (eg, from the EQ-5D-5L) to compare the cost-effectiveness of different interventions.

A key indicator used in a cost utility analysis is QALY, a measure that combines the quantity and quality of health to calculate treatment outcomes that influence health. The cost per QALY for the two treatments will be modeled against work barriers to determine the relative costs and health benefits associated with each treatment. We will use the data from the study to develop a model to estimate the total costs and gains of the intervention in a lifetime perspective.

No interim analyses are planned. Subgroup analyses based on age, gender, the EDSS score, physical activity level at baseline, and geographical region will be performed, as appropriate.

#### Analysis to Handle Protocol Nonadherence and Missing Data

The intention-to-treat principle will be applied to all analyses. Withdrawals from the study, loss to follow-up, and other missing data will be reported, as well as adherence to the intervention as attendance is monitored. In the case of missing data, multiple imputations may be performed, if appropriate.

### Oversight and Monitoring

#### Composition of the Coordinating Center and Trial Steering Committee

The Nordland Hospital Trust is the coordinating center for the trial where the Trial Management Group (TMG) works. The TMG consists of the chief investigator and three study co-investigators who are responsible for the setup of the study, the daily management of the trial, the development of study materials with relevant collaborators, promotion, data management, analysis, and dissemination. The Trial Steering Committee (TSC) consists of neurological consultants with trial experience, statisticians, health economists, quantitative researchers, user representatives, and people with expertise in neurological physiotherapy, cognitive and psychological disabilities in MS, and work adaptations. The TSC will conduct annual meetings to oversee the progress of the trial. Representatives from NAV and four user representatives have been involved in planning and promoting the study, as well as creating materials, such as information videos. The user representatives will take part in discussions throughout the project period, particularly regarding results and implications for practice. The user representatives have participated in both physical and digital meetings and play a central role in delivery of information to members of the MS Association, the MS population, and the general population. Throughout the project, they will find possibilities to impact the project and influence clinical practice within the field.

The study has no data monitoring committee as this is not mandatory for studies not investigating the effects of drugs or medical equipment.

#### Adverse Event Reporting

Any adverse events or harm to the participants during assessments or the intervention will be registered on a case record form and monitored by the TMG. The TMG will keep a log of any negative feedback from the participants. Any serious study-related adverse events will be reported to the regional ethics committee.

The TSC will monitor the progress of the study and adherence to the protocol.

#### Plans for Communicating Important Protocol Amendments to Relevant Parties

Any protocol amendments will be discussed in the TMG and will be made by the chief investigator, who will send any proposed amendments to the regional ethics committee and the data protection officer at the involved study sites for approval. ClinicalTrials.gov will be updated in the case of any major protocol changes. All participants will be notified via email in the case of protocol changes affecting their participation.

### Ethical Considerations

The study has received ethical approval from the Regional Committee for Medical and Health Research Ethics, North (REK-Nord 608117), and the data protection officers at the Nordland Hospital Trust, the University Hospital of North-Norway, the Finnmark Hospital, and the Helgeland Hospital. The study will adhere to the Declaration of Helsinki.

All eligible participants will receive a text message sent to their telephone containing a link to Nettskjema (online form provider) and the TSD’s secure solution for storing research data (developed and hosted by the University of Oslo) containing an information letter and the opportunity to provide consent. Participants will fill out a questionnaire with information about their employment status and digitally sign the consent form. At Nettskjema and TSD login, digital consent is interfaced with the governmental ID portal. The digital consent forms will be stored securely in the TSD database. The TSD is designed for storing and postprocessing sensitive data in compliance with the Norwegian Personal Data Act and Health Research Act. All participants and partners in the project will be treated with respect, and anonymity of participants will be undertaken.

All participants will provide consent to the publishing of anonymous results. No identifying images or other personal information of the participants will be presented. The information videos and exercise booklet contain films and images of models. They have consented to the materials being distributed, as described in the protocol, and in publications from the study. In the case of ancillary studies in the future, ethical approval and separate consent will be obtained.

Dissemination will be conducted in accordance with the Vancouver Protocol. At least two scientific papers will be published in peer-reviewed journals, and results will be presented at national and international meetings and conferences, such as the Norwegian Physiotherapy Association Congress, the World Congress for Physical Therapists, multidisciplinary congresses on MS (eg, Rehabilitation in MS); at meetings in Norwegian and local MS Associations; and in newspapers, podcasts, and websites.

## Results

**Figure 3:**
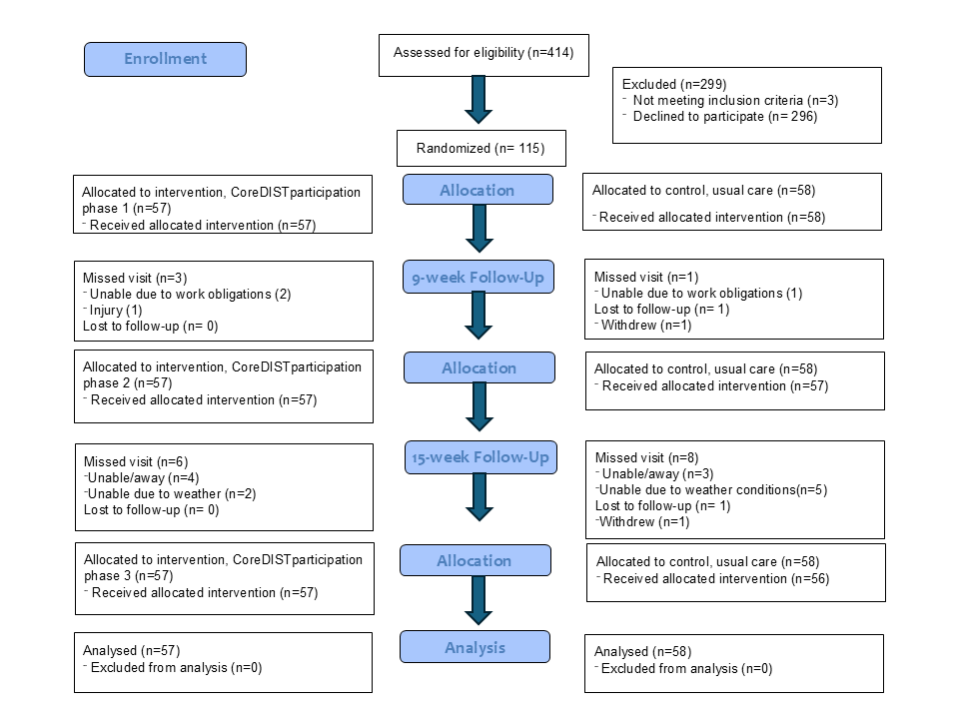
Overview of the flow of participants through the study.

The protocol is currently version 5 as of December 5, 2023. We completed the enrollment phase and enrolled and randomized 115 participants in two phases as of August 1, 2024. The 15-week retests were completed in December 2024, and data collection is estimated to be completed in September 2025. Results are expected to be published in the first quarter of 2026. An overview of the flow of participants through the study is presented in Figure 3.

## Discussion

### Summary

The study will investigate the effectiveness of CoreDISTparticipation, an innovative comprehensive intervention that is the first to integrate the health and welfare sectors by systematically and proactively addressing physical functions, physical activity, and work adaptations. The study includes employed pwMS with minor-to-moderate disability, a group whose vulnerability in terms of early withdrawal from the workforce is well documented [4,5,18,19,27]. During this stage of MS when disability is minor to moderate, the potential for a significant impact at both individual and societal levels is the greatest [17]. Our hypothesis is that compared to usual care, this comprehensive intervention will positively impact the barriers to work experienced, the levels of physical activity, and balance and dynamic trunk control, as well as HRQoL and health economic aspects, such as utility expressed in QALYs and employment status.

The principal findings of potential short- and long-term reduced work-related barriers and work participation following the CoreDISTparticipation intervention will add new knowledge to meet known challenges regarding early withdrawal from the workforce [4,5,18,19,27]. As CoreDISTparticipation includes information videos about the disease and the possibilities for work-related adaptations for both employers and pwMS, as well as a guide for discussion of such challenges with NAV consultants, these measures can easily be implemented in regular welfare in Norway, as well as adapted to similar health and welfare systems. Thus, this study may meet requests from pwMS for adaptations to increase or maintain their employment status [31], which is essential for HRQoL [5].

Given the positive effects of CoreDISTparticipation on physical activity, this study will add valuable information expanding prior work. So far, it has been difficult to document effects on physical activity [58,59], except for an intervention emphasizing behavioral adaptation [60]. If CoreDISTparticipation proves effective compared to usual care, it will point toward a direction for physiotherapy-led activity highlighting a combination of specific core/balance exercises and high-intensity interval training. Moreover, the study will examine fatigue, balance, postural control, walking, and HRQoL—all well-known barriers to work in this population [30,61]—and will provide a deeper understanding of the relationships between these, which can inform health and welfare services.

Finally, this study will provide objective information about health economic factors related to pwMS with low disability. This includes the utility of the CoreDISTparticipation program compared to usual care, expressed in QALYs, long-term employment status, and work-related costs. Given the high work-related costs associated with pwMS [4,7], this study will offer essential insights to further develop cost-effective models of care and potentially enhance workforce sustainability for this group. Thus, the study addresses the part of the follow-up that represents the highest societal costs for pwMS in Norway [4].

If the novel intervention proves effective compared to usual care, it will offer a low-cost approach to be delivered across sectors and health care levels in both rural and urban settings. CoreDISTparticipation can easily be implemented into routine care as it is integrated into existing health and welfare system structures. Educational materials and training programs for NAV consultants and physiotherapists have been developed, and digital materials to optimize physical functions and physical activity for pwMS are being tested in this trial. The exercise component of the intervention requires low-cost equipment that is typically available in standard physiotherapy practices. Moreover, since the trial involves 4 hospital trusts and 22 municipalities of varying sizes in Norway, the necessary flexibility for implementation in both densely and sparsely populated areas will already be covered. CoreDISTparticpation is the first intervention to introduce a proactive approach to well-known work-related challenges experienced by pwMS. The timing of such support, although in active employment, is so far unexplored within the welfare system.

Additionally, the results from the study may be relevant to inform the follow-up care of other groups of young adults with chronic diseases, particularly in the neurological field. The study will provide the health and welfare sectors with new and highly relevant scientific knowledge that could potentially increase sustained employment and physical activity, which may consequently increase health and HRQoL. Such prospects open for future reduced work-related societal costs and increase in the workforce, which is highly requested by authorities [62].

### Limitations

This study is single-blinded as we chose usual care as a comparator and the participants could not be blinded to the treatment they underwent, nor could the physiotherapists who delivered the intervention. The physiotherapists who conducted the testing are, however, blind to allocation.

Although the required number of participants have been recruited successfully, possible issues with implementation fidelity and participant retention may limit the strengths of the study. The physiotherapists involved in the delivery of the intervention have registered attendance and which exercises they have used. These registrations will provide us with insights into whether the intervention has been delivered as intended. In terms of retention, we aim to be as flexible as possible in terms of scheduling of assessments to maintain retention of participants.

Our sample is limited to pwMS who are employed and have an EDSS score of <4. This may limit the ability to make inferences about the feasibility of CoreDISTparticipation for other subgroups of pwMS, such as people who are employed and have an EDDSS score of >4, people who are job seekers, or people who wish to return to the workforce. In addition, the comprehensive nature of the intervention and its focus on physiotherapy and physical activity may attract individuals with an interest in physical activity and may entail fewer participants with more extensive mobility issues or fatigue.

This study will be conducted across a large geographical area, and there is a risk that, for example, the travel distance to test sites, particularly during bad weather conditions in winter, may have introduced attrition bias and will require scrutiny of the characteristics of participants lost to follow up.

## Appendix

Multimedia Appendix 1 SPIRIT checklist.

Multimedia Appendix 2 Description of starting positions for exercises.

Multimedia Appendix 3 Description of all CoreDISTparticipation exercises, objectives, positions, and variations.

Multimedia Appendix 4 Conversation guide for the meeting between the participant and the Norwegian Labour and Welfare Administration (NAV) consultant.

Multimedia Appendix 5 Peer review reports by the Northern Norway Health Authority Research Program scientific evaluation committee.

## Abbreviations

6MWT: 6-minute walk test
COP: center of pressure
EDSS: Extended Disability Status Scale
HRQoL: health-related quality of life
MiniBESTest: Mini Balance Evaluation Systems Test
MS: multiple sclerosis
MSWDQ-23NV: Multiple Sclerosis Work Difficulties Questionnaire-23 – Norwegian version
NAV: Norwegian Labour and Welfare Administration
PROM: patient-reported outcome measure
pwMS: people with multiple sclerosis
QALY: quality-adjusted life-years
RCT: randomized controlled trial
REDCap: Research Electronic Data Capture
TIS-modNV: Trunk Impairment Scale – modified Norwegian version
TMG: Trial Management Group
TSC: Trial Steering Committee
TSD: Tjenester for Sensitive Data (Services for Sensitive Data)
WAA: Work Assessment Allowance
WP: work package

## Data Availability

Key study investigators have access to the final trial dataset. Trial data may be made available to non-study investigators following the approval of a study collaboration request by TSC. There are currently no plans to make the full protocol, participant-level data, and statistical code publicly accessible.

## References

[1] Thompson AJ, Baranzini SE, Geurts J, Hemmer B, Ciccarelli O (2018). Multiple sclerosis. Lancet.

[2] (2023). Epidemiology. MS International Federation.

[3] Aarseth JH, Smedal T, Skår AB, Wergeland S (2023). Annual Report 2022 from the Norwegian MS Register and Biobank.

[4] Skogli E, Halvorsen C, Lønstad C, Vinter C, Hole IH, Stokke OM (2023). Samfunnsøkonomiske konsekvenser av multippel sklerose. MS-forbundet (the MS Association).

[5] Kobelt G, Thompson A, Berg J, Gannedahl M, Eriksson J, MSCOI Study Group, European Multiple Sclerosis Platform (2017). New insights into the burden and costs of multiple sclerosis in Europe. Mult Scler.

[6] Svendsen B, Grytten N, Bø L, Aarseth H, Smedal T, Myhr K (2018). The economic impact of multiple sclerosis to the patients and their families in Norway. Eur J Health Econ.

[7] Johnson Jr WT (2016). The global MS employment report. MS International Federation.

[8] Heine M, van de Port I, Rietberg MB, van Wegen EEH, Kwakkel G (2015). Exercise therapy for fatigue in multiple sclerosis. Cochrane Database Syst Rev.

[9] Razazian N, Kazeminia M, Moayedi H, Daneshkhah A, Shohaimi S, Mohammadi M, Jalali R, Salari N (2020). The impact of physical exercise on the fatigue symptoms in patients with multiple sclerosis: a systematic review and meta-analysis. BMC Neurol.

[10] Learmonth YC, Ensari I, Motl RW (2016). Physiotherapy and walking outcomes in adults with multiple sclerosis: systematic review and meta-analysis. Phys Ther Rev.

[11] Khan F, Amatya B (2017). Rehabilitation in multiple sclerosis: a systematic review of systematic reviews. Arch Phys Med Rehabil.

[12] Arntzen EC, Straume BK, Odeh F, Feys P, Zanaboni P, Normann B (2019). Group-based individualized comprehensive core stability intervention improves balance in persons with multiple sclerosis: a randomized controlled trial. Phys Ther.

[13] Arntzen EC, Straume B, Odeh F, Feys P, Normann B (2020). Group-based, individualized, comprehensive core stability and balance intervention provides immediate and long-term improvements in walking in individuals with multiple sclerosis: a randomized controlled trial. Physiother Res Int.

[14] Gil-González I, Martín-Rodríguez A, Conrad R, Pérez-San-Gregorio MÁ (2020). Quality of life in adults with multiple sclerosis: a systematic review. BMJ Open.

[15] Latimer-Cheung AE, Pilutti LA, Hicks AL, Martin Ginis KA, Fenuta AM, MacKibbon KA, Motl RW (2013). Effects of exercise training on fitness, mobility, fatigue, and health-related quality of life among adults with multiple sclerosis: a systematic review to inform guideline development. Arch Phys Med Rehabil.

[16] Rooney S, Riemenschneider M, Dalgas U, Jørgensen M-LK, Michelsen A, Brønd JC, Hvid LG (2021). Physical activity is associated with neuromuscular and physical function in patients with multiple sclerosis independent of disease severity. Disabil Rehabil.

[17] Tomassini V, Matthews PM, Thompson AJ, Fuglø D, Geurts JJ, Johansen-Berg H, Jones DK, Rocca MA, Wise RG, Barkhof F, Palace J (2012). Neuroplasticity and functional recovery in multiple sclerosis. Nat Rev Neurol.

[18] Bøe Lunde HM, Telstad W, Grytten N, Kyte L, Aarseth J, Myhr K, Bø L (2014). Employment among patients with multiple sclerosis-a population study. PLoS One.

[19] Glad SB, Nyland H, Aarseth JH, Riise T, Myhr K (2011). How long can you keep working with benign multiple sclerosis?. J Neurol Neurosurg Psychiatry.

[20] Simmons RD, Tribe KL, McDonald EA (2010). Living with multiple sclerosis: longitudinal changes in employment and the importance of symptom management. J Neurol.

[21] Clemens L, Langdon D (2018). How does cognition relate to employment in multiple sclerosis? A systematic review. Mult Scler Relat Disord.

[22] Grytten N, Skår AB, Aarseth JH, Assmus J, Farbu E, Lode K, Nyland HI, Smedal T, Myhr KM (2017). The influence of coping styles on long-term employment in multiple sclerosis: a prospective study. Mult Scler.

[23] Kavaliunas A, Wiberg M, Tinghög P, Glaser A, Gyllensten H, Alexanderson K, Hillert J (2015). Earnings and financial compensation from social security systems correlate strongly with disability for multiple sclerosis patients. PLoS One.

[24] Guerra T, Pipoli A, Viterbo RG, Manghisi N, Paolicelli D, Iaffaldano P, Di Lorenzo L (2022). Predictors of unemployment status in people with relapsing multiple sclerosis: a single center experience. Neurol Sci.

[25] Julian LJ, Vella L, Vollmer T, Hadjimichael O, Mohr DC (2008). Employment in multiple sclerosis. Exiting and re-entering the work force. J Neurol.

[26] Bogenschutz M, Rumrill Jr PD, Seward HE, Inge KJ, Hinterlong PC (2016). Barriers to and facilitators of employment among Americans with multiple sclerosis: results of a qualitative focus group study. J Rehabil.

[27] Salter A, Thomas N, Tyry T, Cutter G, Marrie RA (2017). Employment and absenteeism in working-age persons with multiple sclerosis. J Med Econ.

[28] Krause JS, Dismuke-Greer CL, Rumrill P, Reed K, Jarnecke M, Backus D (2022). Job retention among individuals with multiple sclerosis: relationship with prediagnostic employment and education; demographic characteristics; and disease course, severity, and complications. Arch Phys Med Rehabil.

[29] Emery H, Padgett C, Ownsworth T, Honan CA (2023). "Oh it's changed, it's changed 10-fold": understanding the experience of self-concept change from the perspectives of people with multiple sclerosis. Disabil Rehabil.

[30] Raggi A, Covelli V, Schiavolin S, Scaratti C, Leonardi M, Willems M (2016). Work-related problems in multiple sclerosis: a literature review on its associates and determinants. Disabil Rehabil.

[31] Arntzen EC, Braaten T, Fikke HK, Normann B (2023). Feasibility of a new intervention addressing group-based balance and high-intensity training, physical activity, and employment in individuals with multiple sclerosis: a pilot randomized controlled trial. Front Rehabil Sci.

[32] Fikke HK, Norman B, Sivertsen M, Dahl SSH, Arntzen EC (2022). Utprøving av intervensjon for optimalisering av funksjon, fysisk aktivitet og arbeidsdeltakelse ved multippel sklerose. Fysioterapeuten.

[33] Cohen N, Arnold G, Petridou E (2023). Why we need to study street‐level policy entrepreneurs. Eur Policy Anal.

[34] NAV dialog meeting. Norwegian Labour and Welfare Administration.

[35] Normann B, Zanaboni P, Arntzen EC, Øberg GK (2016). Innovative physiotherapy and continuity of care in people with multiplesclerosis: a randomized controlled trial and a qualitative study. J Clin Trials.

[36] Lahelle AF, Øberg GK, Normann B (2020). Group dynamics in a group-based, individualized physiotherapy intervention for people with multiple sclerosis: a qualitative study. Physiother Res Int.

[37] Lahelle AF, Øberg GK, Normann B (2020). Physiotherapy assessment of individuals with multiple sclerosis prior to a group intervention - a qualitative observational and interview study. Physiother Theory Pract.

[38] Lahelle AF, Øberg GK, Normann B (2018). A group-based, individualized physiotherapy intervention for people with multiple sclerosis-a qualitative study. Physiother Res Int.

[39] Arntzen EC, Øberg GK, Gallagher S, Normann B (2021). Group-based, individualized exercises can provide perceived bodily changes and strengthen aspects of self in individuals with MS: a qualitative interview study. Physiother Theory Pract.

[40] Dahl SSH, Arntzen EC, Normann B (2024). The meaningfulness of through interactions and enjoyment in outdoor high-intensity physiotherapy for people with multiple sclerosis: a qualitative study. Front Rehabil Sci.

[41] McDonald WI, Compston A, Edan G, Goodkin D, Hartung HP, Lublin FD, McFarland HF, Paty DW, Polman CH, Reingold SC, Sandberg-Wollheim M, Sibley W, Thompson A, van den Noort S, Weinshenker BY, Wolinsky JS (2001). Recommended diagnostic criteria for multiple sclerosis: guidelines from the International Panel on the Diagnosis of Multiple Sclerosis. Ann Neurol.

[42] Forskningsprosjekt. CoreDIST. Nord universitet.

[43] Raine S, Raine S, Meadows L, Lynch-Ellenington M (2009). The Bobath concept: developments and current theoretical understanding. Bobath Concept: Theory and Clinical Practice in Neurological Rehabilitation.

[44] Shumway-Cook A, Woollacott MH, Rachwani J, Santamaria V (2023). Motor Control: Translating Research into Clinical Practice.

[45] Youssef H, Gönül MN, Sobeeh MG, Akar K, Feys P, Cuypers K, Vural A (2024). Is high-intensity interval training more effective than moderate continuous training in rehabilitation of multiple sclerosis: a comprehensive systematic review and meta-analysis. Arch Phys Med Rehabil.

[46] Honan CA, Brown RF, Hine DW (2014). The multiple sclerosis work difficulties questionnaire (MSWDQ): development of a shortened scale. Disabil Rehabil.

[47] Block VAJ, Pitsch E, Tahir P, Cree BAC, Allen DD, Gelfand JM (2016). Remote physical activity monitoring in neurological disease: a systematic review. PLoS One.

[48] Bennett SE, Bromley LE, Fisher NM, Tomita MR, Niewczyk P (2017). Validity and reliability of four clinical gait measures in patients with multiple sclerosis. Int J MS Care.

[49] Feng Y, Kohlmann T, Janssen MF, Buchholz I (2021). Psychometric properties of the EQ-5D-5L: a systematic review of the literature. Qual Life Res.

[50] Lerdal A, Johansson S, Kottorp A, von Koch L (2010). Psychometric properties of the Fatigue Severity Scale: Rasch analyses of responses in a Norwegian and a Swedish MS cohort. Mult Scler.

[51] McGuigan C, Hutchinson M (2004). Confirming the validity and responsiveness of the Multiple Sclerosis Walking Scale-12 (MSWS-12). Neurology.

[52] Hamre C, Botolfsen P, Tangen GG, Helbostad JL (2017). Interrater and test-retest reliability and validity of the Norwegian version of the BESTest and mini-BESTest in people with increased risk of falling. BMC Geriatr.

[53] Gjelsvik B, Breivik K, Verheyden G, Smedal T, Hofstad H, Strand LI (2012). The Trunk Impairment Scale - modified to ordinal scales in the Norwegian version. Disabil Rehabil.

[54] Weikert M, Motl RW, Suh Y, McAuley E, Wynn D (2010). Accelerometry in persons with multiple sclerosis: measurement of physical activity or walking mobility?. J Neurol Sci.

[55] Smedal T, Johansen HH, Myhr K-M, Strand LI (2010). Psychometric properties of a Norwegian version of Multiple Sclerosis Impact Scale (MSIS-29). Acta Neurol Scand.

[56] Farrar JT, Young JP, LaMoreaux L, Werth JL, Poole MR (2001). Clinical importance of changes in chronic pain intensity measured on an 11-point numerical pain rating scale. Pain.

[57] Harris PA, Taylor R, Thielke R, Payne J, Gonzalez N, Conde JG (2009). Research Electronic Data Capture (REDCap)--a metadata-driven methodology and workflow process for providing translational research informatics support. J Biomed Inform.

[58] Motl RW, Sandroff BM, Kwakkel G, Dalgas U, Feinstein A, Heesen C, Feys P, Thompson AJ (2017). Exercise in patients with multiple sclerosis. Lancet Neurol.

[59] Arntzen EC, Bidhendi-Yarandi R, Sivertsen M, Knutsen K, Dahl SSH, Hartvedt MG, Normann B, Behboudi-Gandevani S (2023). The effect of exercise and physical activity-interventions on step count and intensity level in individuals with multiple sclerosis: a systematic review and meta-analysis of randomized controlled trials. Front Sports Act Living.

[60] Motl RW, Kidwell-Chandler A, Sandroff BM, Pilutti LA, Cutter GR, Aldunate R, Bollaert RE (2023). Randomized controlled trial of the behavioral intervention for physical activity in multiple sclerosis project: social cognitive theory variables as mediators. Mult Scler Relat Disord.

[61] (2018). EQ-5D-3L user guide. EuroQol Research Foundation.

[62] Myklathun KH, Skjøstad O (2024). NAVs BEDRIFTSUNDERSØKING 2024: Redusert mangel på arbeidskraft (NAV's Business survey 2024: less shortage of labour). Norwegian Labour and Welfare Administration.

