# Supplementary material for: A Comprehensive and Structured Follow-Up for Persons With Multiple Sclerosis (CoreDISTparticipation) to Optimize Physical Functions, Health, and Employment: Protocol for a Prospective, Single-Blinded Randomized Controlled Trial and Health Economic Evaluation
Source: JMIR Res Protoc. 2025 Oct 8;14:e74988. doi: 10.2196/74988 (PMC12547332; doi:10.2196/74988)
Supplement: Multimedia Appendix 2 [file resprot_v14i1e74988_app2.pdf]

Supplementary file 2: Description of starting positions of the exercises.

STARTING POSITION	DESCRIPTION
Prone	Keep both feet in contact with the wall, toes pointing down, the body and hips in contact with the bench/floor. Keep a straight line between the ankles, knees, hips, trunk, shoulders 90° abducted, elbows and wrist extended, and the palms facing down. You may rotate your neck/head to one side or face down.
Supine	Keep both feet in contact with the wall, toes pointing up, keep the back in contact with the bench/floor. Keep a straight line between the ankles, knees and hips, the back, thorax and neck, and the elbows, hands and fingers extended.
Supine with therapy ball under lumbar back/pelvis	Keep both feet on the floor, the back and pelvic on the therapy ball and the thorax, head and arms on the bench. Keep a straight line between the ankles, knees and hips.
Sidelying	Keep a straight line throughout the body, both feet placed on the wall, hip-width apart. The arms may point forward with 90°flexion in the shoulders and the elbows, hands and fingers extended, for instance pushing a spiky ball forward.
Four-point kneeling	Keep the therapy ball under your stomach, both feet, ankles, knees and hips in a straight line, hip-width apart, toes pointing backwards. Back and head in a straight line. Extended elbows, hands and fingers, hands placed on the bench.
High kneeling	Kneeling position with hips extended and trunk upright.
Half kneeling	Keep one hip extended, with weightbearing at the knee and upper leg. The opposite hip and knee are flexed to approximately 90 degrees, weightbearing on the foot placed forward on the floor.
Sitting	Keep an upright position with the trunk and neck, both feet on the floor and a straight line between the ankles, knees and hips. Keep the lumbar back actively in contact with the therapy ball. The hands placed beside you on the bench with shoulders external rotated, elbows, hands and fingers extended.
Sitting with feet on the bench	Bend the hips and knees, feet placed on the bench. Keep the back actively in contact with a therapy ball.
Sitting on heels	Keep the bottom as far down towards the heels as you can, toes pointing backwards. Keep a straight line from the lumbar back to the neck.
Standing	Keep an upright position with the back, thorax and neck in a straight line and the feet hip-width apart, toes pointing forward with a straight line between the ankles, knees and hips. Shoulders externally rotated, elbows, hands and fingers extended.

Standing with the therapy ball behind you or in front of you	Keep an upright position with the back, thorax and neck in a straight line and the feet hip-width apart, toes pointing forward with a straight line between the ankles, knees and hips. Keep the lumbar back actively in contact with the therapy ball or you keep the therapy ball actively between the abdomen and the wall in front of you. Shoulders externally rotated, elbows, hands and fingers extended placed on the wall.
” Bear-standing”	Standing in a flexed position with both feet and hands placed on the floor, hip-/shoulder-width apart. Keep a straight line between the ankles, knees and hips. The elbows, hands and fingers extended.
