# Supplementary material for: A Comprehensive and Structured Follow-Up for Persons With Multiple Sclerosis (CoreDISTparticipation) to Optimize Physical Functions, Health, and Employment: Protocol for a Prospective, Single-Blinded Randomized Controlled Trial and Health Economic Evaluation
Source: JMIR Res Protoc. 2025 Oct 8;14:e74988. doi: 10.2196/74988 (PMC12547332; doi:10.2196/74988)
Supplement: Multimedia Appendix 3 [file resprot_v14i1e74988_app3.pdf]

Supplementary file 3: Descriptions of all CoreDISTparticipation exercises, objectives, positions and variations (various levels of difficulty). All exercises can be conducted with manual facilitation and tissue mobilization. In all the exercises dual or multiple task challenges can be added to increase the level of difficulty. The exercises can be conducted with eyes open or closed and on even or uneven surface. Most of the exercises can be conducted both in an in-door and outdoor environment and performed with low, moderate and high intensity by increasing speed.

Supplementary file 2a Pre- and post-training balance self-assessment.

NR	EXERCISE	OBJECTIVE	POSITION	VARIATIONS
	**Pre- and post-training balance self-assessment**	Exploring own limits for balance before and after each training session.	Standing<br><br>These self-assessments can be conducted with eyes open or closed, standing on even or uneven surface.	1) Roll from toe to heel and back again.<br>2) Roll from lateral to medial edge of the foot and back again.<br>3) "The Kibler", inspired by Kibler, Press & Sciascia, 2006 (1)<br>a) Lift the forefoot and outer board of one foot.<br>b) Keep the position while lifting the heal of the floor, weight transferring to the opposite leg.<br>c) While standing on one leg, bend the opposite knee and push the heal backwards extending the hip.<br>d) Keep the position while bending down on the standing leg (one leg squat).<br>e) Touch the floor with the opposite hand.<br>f) Bend the neck.<br>e) Slowly roll back to a standing position.

Supplementary file 2b: Exercises for sensorimotor activation

NR	EXERCISE	OBJECTIVE	POSITION	VARIATIONS
1	Rolling a small mobilization ball with one foot	Core activation while sensory motor activation of the foot	Sitting	Maintain the position by core activation while rolling the ball with one foot:<br>1) back and forward.<br>2) with the heel only.<br>3) with the forefoot only.<br>Keep the heel stable toward the floor:<br>4) roll the ball with the forefoot in lateral-medial direction.<br>5) flex and extend/abduct the toes.
2	Rolling a small mobilization ball with the hands	Core activation while sensory motor activation of the hands and fingers.	Sitting, standing, standing with a therapy ball behind	Maintain the position by core activation while:<br>1) rolling a spiky ball with your hands on the bench far back and forward.<br>2) rolling a spiky ball with your hands on the bench far back, keep the position while conducting opposition movements with your fingers.<br>3) rolling a spiky ball between your hands as far back and forward as you can with extended arms.<br>4) holding a spiky ball in front of you between your extended hands/fingers and another between your 90° flexed elbows, then, roll the ball in your hands.
3	Oculomotor and vestibular exercises	Core activation while visual fixation, combined with head, neck and tracking movements	Any of the described starting positions or in combination with other exercises.	Maintain the position by core activation and:<br>1) Focus on one point in front of you while moving the head in rotation, lateral flexion or up-down.<br>2) Push the spiky ball forward with extended arms. Hold the ball still and maintain visual fixation on the ball while moving the head; rotation, lateral flexion or up-down.<br>3) Hold your head still, nose pointing forward and keep visual fixation on the ball while moving it from side to side.<br>4) Hold one ball in each hand with extended and abducted arms. Look at one ball and then direct vision towards the other without turning your head. Afterwards you may turn your head and repeat to the opposite side.

Supplementary file 2c: Exercises for improving muscle length, mobility and coordination

NR	EXERCISE	OBJECTIVE	POSITION	VARIATIONS
4	"Bent banana" and "Straight banana"	Core activation while concentric activation of the hip- and knee flexors, and eccentric lengthening of the knee- and hip extensors	Supine	Maintain the position by core activation while:<br>1) bending one knee and pulling it toward the opposite shoulder while holding your hands around the knee.<br>2) bending the knee without holding it.<br>3) repeating the same exercises with your arms and hands extended, pointing in front of you or keeping your arms abducted.<br>4) elevating the leg, heel pointing towards the ceiling and toe pointing towards the opposite shoulder.
5	"The eagle"	Core activation while concentric activation of hip extensors and knee flexors, and eccentric lengthening of the hip flexors and knee extensors.	Prone	Maintain the position by core activation while:<br>1) bending one knee and pushing the heel toward the ceiling.<br>2) bending one knee and pushing the heel toward the ceiling, followed by pushing the heel towards the opposite hip and then towards the ceiling.<br>3) lifting the opposite arm and leg (diagonal extension)<br>4) lifting the opposite arm and leg (diagonal extension) while lifting both the head and both arms from the bench.
6	"Slowly diving"	Core activation while gradually eccentric lengthening of upper trunk and neck extensors.	Sitting or standing with the ball behind you.	Maintain the position by core activation while:<br>1) rolling down towards a foam block or the floor, in sitting.<br>2) rolling down towards a foam block, in standing. Bend the knees and posterior tilt the pelvis while rolling up again.<br>3) rolling down towards the floor, therapy ball behind your back.<br>4) rolling down towards the floor without the therapy ball.
7	"Butterfly"	Core activation while concentric work in the trunk and neck extensors, and eccentric lengthening of the abdominal, thorax, arms and neck flexors.	Sitting or standing with the ball behind you or prone lying with the ball under you	Maintain the position by core activation while:<br>1) leaning halfway back and abducting the arms 45°, sitting position with the hips and knees flexed, feet on the bench.<br>2) leaning halfway back and abducting the arms 45°, sitting position.<br>3) leaning as far back as you can without losing contact with the ball.<br>4) leaning all the way back and abducting the shoulders and extending the arms.

8	"Pluto"	Core activation while eccentric lengthening the hip, back-and neck extensors	Sitting on heals	Maintain the position by core activation while:<br>1) rolling a large therapy ball forward extending your arms and hands.<br>2) reaching your hands and stretching your arms as far as you can straight forward on the bench/floor or while rolling two spiky balls forward.<br>3) reaching with the arms forward on the floor/bench or by rolling spiky balls.<br>4) reaching one arm and hand under the other and rotating the thorax.
9	"Nodding"	Core activation while lengthening neck extensors and lateral flexors.	Sitting, sitting with flexed feet or standing, hands on the bench.	Maintain the position by core activation while:<br>1) nodding straight forward and sideways.<br>2) lateral flexing the head and then rotating the head, nose pointing at the shoulder.<br>3) lateral flexing the head and pushing the opposite arm towards the bench.<br>4) rotating the head to the left, nodding to the person next to you, repeat to the opposite side.

Supplementary file 2d: Exercises in lying positions for selective movement control and coordination in the trunk, pelvis, hips and shoulders

NR	EXERCISE	OBJECTIVE	POSITION	VARIATIONS
10	"Standing- on the wall"	Core activation while keeping one or two legs stable and moving the hip into flexion and extension.	Supine	Maintain the position by core activation while:<br>1) keeping both feet on the wall, pushing both heels towards the wall and stand straight.<br>2) keeping one foot in contact with the wall and bending the other leg, foot on the bench. Extend the hip by pushing the knee forward.<br>3) keeping one foot in contact with the wall and bend the other leg, foot on the bench. Roll a spiky ball with your foot on the bench.<br>4) keeping one foot in contact with the wall, lifting the opposite leg, flexing the knee towards your belly and extending it toward the bench.
11	"One leg balancing on the wall"	Core activation while keeping one leg stable towards the wall and moving the opposite leg, extending or abducting the hip.	Supine	Maintain the position by core activation while:<br>1) bending the hip and knee, place the foot on the bench and then slowly move the knee to the side while keeping the other hip/pelvis stable. Return to center.<br>2) placing one foot on a foam block placed on the floor, extending the hip and flexing the knee. Then lift the outer edge of the foot and push the heal towards the box.<br>3) placing one foot on a foam block placed on the floor, then slowly move the knee to the side while keeping the other hip/pelvis stable. Return to center.<br>4) placing one foot on the floor, then push the heel towards the floor and move the knee to the side. Return to the center.
12	"Walking on the wall"	Core activation while keeping one or two legs stable and moving the hip into flexion and extension. Coordinated movements with the arms.	Supine	Maintain the position by core activation while:<br>1) walking on the wall while pushing each foot towards the wall and both arms toward your toes.<br>2) lifting the lateral surface of the feet and pushing each arm towards your toes.<br>3) bending your knees and pushing each knee forward extending every other hip. Push the opposite arm and hands forward simultaneously.<br>4) walking on the wall with extended legs while pushing the opposite arm and hand towards the toes.
13	"The spider"	Optimal contact with the base of support. Active and	Supine with your hips and knees	Maintain the position by core activation while:<br>1) tilting the pelvis in a-p direction over a rolled towel

		stable core in combination with pelvic movements and activation of muscles in the back, buttocks and posterior thigh.	flexed, and the feet placed on the bench or the wall.	2) performing the same movement as 1 while pushing the arms and hands towards the toes.<br>3) bending at the hips and knees and placing them on a psoas pillow placed on the bench, feet in contact with the wall. Perform pelvic tilts in a-p direction over a rolled towel.<br>4) bending at the hips and knees and placing your heels on a large therapy ball. Pelvic tilts in a-p direction over a rolled towel.
14	"The shrimp"	Core activation, keeping the upper part of the thorax stable while moving the lower abdomen, hips and both legs.	Supine with flexion in hips and knees, heels on the therapy ball. Keep a straight line between the ankles, knees and hips.	Maintain the position by core activation while:<br>1) Keeping one leg stable (foot on the bench) and rolling the large therapy ball back and forward.<br>2) Placing both heels on the ball and rolling it back and forward.<br>3) Rolling the ball from side to side, arms abducted on the bench.<br>4) Rolling the ball from side to side while pushing a small ball with your hands in the direction of the knees.
15	"The crab-wiggles and plays with the ball"	Core activation while keeping both legs stable and moving the pelvis, arms and shoulders.	supine with the therapy ball under lumbar back/pelvis.	Maintain the position by core activation while:<br>1) rolling the ball up and down (tilt the pelvis in anterior-posterior direction) and from side to side. Arms abducted on the bench.<br>2) Same movements as 1 while pushing the arms and hands towards the toes. Extend one leg while conducting the movement.<br>3) conducting "angel wings" with your arms and clap your hands.<br>4) throwing the ball up and catching it (or play together with the therapist).
16	"The cat"	Core activation while keeping both legs and arms stable and moving the pelvis or thorax.	Four-point kneeling	Maintain the position by core activation while:<br>1) performing pelvic tilts in a-p direction while simultaneously extending and flexing the thorax and the column.<br>2) keeping the shoulders and thorax still while performing pelvic tilts in a-p direction. Then keep the pelvis in a neutral position and retract-protract the shoulders.

				3) keeping a neutral position of the back and pelvis while extending one leg, pushing the heel toward the ceiling. Release and lift the other leg.<br>4) keeping a neutral position of the back and pelvis while extending one leg and the opposite arm, thumb and heel toward the ceiling. Release and repeat on the opposite side.
17	"The stick"	Core activation while keeping both arms and one or two legs stable and moving the hips and legs.	Side-lying	Maintain the position by core activation while:<br>1) pushing your extended arms towards the wall, release and retract the shoulders. Repeat.<br>2) placing the top leg on a large therapy ball behind you and roll the ball back and forward using the upper leg (hip extension).<br>3) bending the upper knee, placing the leg on a psoas pillow or the large ball, and lifting the knee towards the ceiling (hip abduction), release.<br>4) bending the upper knee, placings the leg on a psoas pillow or the large ball and abducting the upper arm and rotate the upper body so that the arm is stretched behind you. Release and move the arm back to the wall in front of you.
18	"Sit-up"	Active and stable core in combination with transfer from supine to sitting	Supine	Maintain the position (both feet in contact with the wall) by core activation while:<br>1) rolling half-way up from lying and back again, pushing the arms towards the toes (or pushing a ball forward).<br>2) rolling all the way up to a sitting position, pushing the arms towards the toes (or pushing a ball forward).<br>3) keeping one leg in contact with the wall and the other on the floor while rolling half-way up from lying and back again, pushing the arms towards the toes (or pushing a ball forward).<br>4) keeping one leg in contact with the wall and the other on the floor while rolling all the way up from lying and back again, pushing the arms towards the toes (or pushing a ball forward).

Supplementary file 2e: Exercises in sitting for selective movement control and coordination in the trunk, pelvis, hips and shoulders

NR	EXERCISE	OBJECTIVE	POSITION	VARIATIONS
19	"Hands up"	Core activation, keeping the hips and legs stable on the bench while moving the arms and the trunk.	Sitting or standing	Maintain the position by core activation while:<br>1) pushing the heel of the hand towards the bench. Release and elevate the shoulders.<br>2) bending both elbows and pushing them towards the bench and reaching one elbow towards the bench while lateral flexing the trunk. Return to up-right position.<br>3) holding a towel or ball between your elbows and pushing the elbows forward, release and retract the shoulders.<br>4) holding a towel or ball between your elbows and pushing the elbows forward while rotating arms and trunk toward one side. Return to the center. Repeat to the opposite side.
e20	"Rolling the ball"	Core activation while keeping both legs stable and moving the pelvis.	Sitting or standing	Maintain the position by core activation while:<br>1) pushing the heel of the hands towards the bench and rolling the ball back and forward (a-p tilt of the pelvis).<br>2) pushing the heel of the hands towards the bench and rolling the ball from side to side (lateral weight transfer).<br>3) conducting number 1 and 2 while pushing your arms forward.<br>4) rolling the ball to one side and delivering a towel to the therapist. Repeat to the opposite side.
21	"Pelvic walk"	Active and stable core in combination with active rotations of the lower trunk	Sitting	Maintain the position by core activation while:<br>1) pushing each knee forward extending the hip while keeping the upper trunk still.<br>2) pushing each knee forward towards a large therapy ball while keeping the upper trunk still.<br>3) conducting 1 or 2 while pushing both hands forward, extending the elbows.<br>4) conducting 1 or 2 while pushing the opposite arm forward, extending the elbows.
22	"The angel"	Core activation while keeping both legs stable and moving the arms and shoulders.	Sitting or standing	Maintain the position by core activation while:<br>1) retracting and protracting the shoulders, keeping the hands in contact with the bench.<br>2) retracting the shoulders and abducting the arms as far back as you can without elevating the rib cage.<br>3) conducting nr. 2 and making "angel wings".<br>4) conducting nr. 3 and raise to standing on your toes.

23	"Reach all over and pick berries"	Ability to move the center of gravity forward and sideways within and outside the base of support. Active and stable core in combination with larger muscle groups in the legs.	Sitting or standing	Maintain the position by core activation while:<br>1) lateral flexing the trunk to reach an object placed next to you.<br>2) leaning forward to reach an object placed near your opposite foot.<br>3) reaching for an object placed next to you or behind you.<br>4) In standing: taking a step forward and pick up an object lying on the ground using opposite arm and leg.

Supplementary file 2f: Exercises in standing for trunk control in coordination with larger muscle groups

NR	EXERCISE	OBJECTIVE	POSITION	VARIATIONS
24	“High kneeling”	Active and stable core in combination with lengthening of the hip flexors, lateral weight shift and arm movements	High kneeling with the large ball in front of you	Maintain the position by core activation while:<br>1) extending your hips and pushing the ball forward towards the wall or the therapist. Release and sit down towards the heels.<br>2) keeping the extended position, holding the ball towards the wall and rolling the ball from side to side (lateral weight transfer).<br>3) conducting number 3 while elevating the arm high up towards the wall in the same direction.<br>4) placing one foot on the floor (half kneeling), keeping the ball still by extending your hip and then moving the bended knee forward and outwards.
25	“Squats”	Core activation, keeping the upper part of the thorax and the feet stable while moving the pelvis, hips and knees.	Standing with the therapy ball behind your back.	Maintain the position by core activation while conducting regular squats simultaneously as you:<br>1) roll the ball up and down on the wall behind you. Before you stand up- posterior tilt the pelvis and keep the hip extensor muscles activated while extending to an upright position.<br>2) roll the ball up and down on the wall behind you while pushing a ball forward with your hands. Before you stand up- posterior tilt the pelvis and hold the hip extensor activated while extending to an upright position.<br>3) repeat number 2 while bending your elbows, holding a ball between your elbows and pushing them forward.<br>4) The same exercise may be conducted with a) a large therapy ball in front of you or b) both in front and behind you or c) leaning forward towards a ball on the bench in front of you or d) with no support.
26	“Squats on one leg”	Core activation, keeping the upper part of the thorax and one leg stable while moving the pelvis, hip and knee.	Standing with the therapy ball in front of your stomach.	Maintain the position by core activation while conducting squats:<br>1) on both legs, keeping the hands placed right in front of you on the wall.<br>2) on one leg, pushing the hands forward towards the wall with extended arms.<br>3) on one leg, hands placed on the wall and the opposite foot placed on a foam block.<br>4) on one leg, hands placed on the wall and the opposite foot pushing the foam block backwards extending the hip.

27	"The bear squats"	Core activation while keeping the upper part of the thorax, arms and one or two feet stable while moving the pelvis, hips and knees.	Bear-standing	Maintain the position by core activation while:<br>1) placing your hands on a bench or chair in front of you, extended and activated arms while conducting squats.<br>2) placing your hands on the floor, extended and activated arms while conducting squats.<br>3) keeping the same position as in number 2, lifting one leg, heel pointing towards the ceiling, and conducting squats on one leg.<br>4) keeping the same position, lifting one leg and the opposite arm and conducting squats on one leg.
28	"The bear calf-rise"	Core activation while keeping the upper part of the thorax, arms and one or two feet stable while moving the pelvis, ankle, foot and calf.	Bear-standing	Maintain the position by core activation while:<br>1) placing your hands on a bench or chair in front of you, extended and activated arms while conducting calf-rise.<br>2) placing your hands on the floor, extended and activated arms while conducting calf-rise.<br>3) keeping the same position as in number 2, lifting one leg and conducting calf-rise on one leg.<br>4) keeping the same position, lifting one leg and the opposite arm and conducting calf-rise on one leg.
29	"Calf-rise"	Core activation while keeping the upper part of the thorax, arms and one or two feet stable while moving the pelvis, and ankle, foot and calf.	Standing with the therapy ball in front of your stomach. Hands placed on the wall.	Maintain the position by core activation while conducting calf-rise:<br>1) on both legs, keeping the hands placed right in front of you on the wall.<br>2) on one leg, pushing the hands forward towards the wall with extended arms.<br>3) on one leg, hands placed on the wall and the opposite foot placed on a foam block.<br>4) on one leg, hands placed on the wall and the opposite foot pushing the foam block backwards, extending the hip.
31	"The pole"	Core activation while keeping the upper part of the thorax, arms and one or both feet stable while	Standing with the therapy ball in front of you. Shoulders, elbows,	Maintain the position by core activation while:<br>1) retracting and protracting the shoulders. Hold the retracted position while conducting pelvic tilts in a-p direction.<br>2) retracting the shoulders and conducting calf-rise.<br>3) retracting the shoulders, posterior tilting the pelvis and conducting calf-rise.<br>4) lifting one leg from the floor and conduct calf-rise on one leg.

		moving the shoulders, pelvis or ankle.	hands and fingers, extended. Hands placed on a bench behind you.	
31	"The march"	Core activation, keeping the back and the upper part of the thorax stable while moving the ankles, knees and hips.	Standing with the ball behind your back.	Maintain the position by core activation while:<br>1) lifting the lateral surface of one foot and stomping to a beat.<br>2) pushing your arms forward (holding a ball) and stomping to the beat, one leg at the time (lateral weight transfer from one leg to the other).<br>3) pushing the ball forward with extended arms. March while lifting your knees high. Can also be conducted while kicking your heels back<br>4) The exercise can also be conducted without the large ball behind you back and with increased pace (running).
33	"The hip-wiggle"	Core activation, keeping the upper part of the thorax, arms and one or two feet stable while moving the pelvis, hips and ankle.	Standing with the therapy ball in front of you, hands on the wall. Place one foot on a chair in front of you.	Keep the position by core activation while:<br>1) lifting the large ball with your stomach, posterior tilting the pelvis. Release controlled.<br>2) pushing the knee of the leg that is placed on the chair forward by activating the hip extensors on the standing leg, then move the knee laterally and return to forward pointing position (hip-hitching).<br>3) conducting calf-rise on one leg.<br>4) conducting calf-rise on one leg, clap your hands when you are in the highest position.<br>The exercise can be conducted with a therapist sitting in-front.

Supplementary file 2g: Advanced postural control and balance exercises

NR	EXERCISE	OBJECTIVE	POSITION	VARIATIONS
33	"The ball-play"	Core activation while keeping the back and lower part of the thorax and abdomen stable while moving the arms.	Standing with the ball behind you or with no support.	Maintain the position by core activation while:<br>1) throwing a ball, bean-bag or towel with the therapist or the others in the group.<br>2) pushing a weight ball forward or throwing it sideways to the therapist or the others in the group.<br>3) throwing a weight ball over your head and bounce it into the ground to the therapist or the others in the group.<br>4) throwing more than one ball, bean-bag, towards etc. to each other in the group.
34	"The bounce"	Core activation while moving both arms and legs.	Standing	Maintain the position by core activation while:<br>1) bouncing the ball into the ground and catching it again.<br>2) bouncing the ball while doing squats and/or calf-rise.<br>3) bouncing the ball using each hand.<br>4) bouncing while jumping.
35	"The waiter"	Core activation while keeping the feet, the back and the thorax stable while rotating and/or extending the trunk.	Standing with the ball behind you or back-to-back with another participant in the group.	Maintain the position by core activation while:<br>1) keeping your back towards a large therapy ball or participant's back. Extend your arms, hold a ball in your hands. Rotate the body and deliver the ball as far back to the wall or to the partner standing behind you.<br>2) Same position as nr. 1. Deliver the ball over your head while extending your back and elevating the arms.<br>3) Standing back-to-back with another participant. Deliver the ball between your legs.<br>4) Combine 1, 2 and 3.
36	Run away	Core activation while performing gait and running movements and increasing pulse.	Standing with a rubber band held tight around your belly. The rubber band may be attached to	Maintain the position by core activation while:<br>1) marching fast on the spot while the therapist or another participant holds a rubber band tight around your belly.<br>2) holding a spiky ball in your hands and pushing it forward with extended elbows. March fast on the spot while lifting your knees high. The therapist or another participant holds a rubber band tight around your belly.<br>3) walking or running forward while the therapist or another participant holds a rubber band tight around your belly to provide resistance.

			the doorknob, a three or can be held by the therapist or participant.	4) jumping up and down or conducting scissor jumps while the therapist or another participant holds a rubber band tight around your belly.
37	"Step up"	Core activation while performing step up movements and increasing pulse.	Standing with a rubber band held tight around your belly. The rubber band may be attached to the doorknob, a three or be hold by the therapist or other participant.	Maintain the position by core activation while:<br>1) going up and down on a step or stepping box while having a rubber band tight around your belly.<br>2) taking a step sideways on a step or box while having a rubber band tight around your belly.<br>3) running up and down on a step or box while having a rubber band tight around your belly.<br>4) performing the exercise without the rubber band.

Supplementary file 2h: Relaxation and self-massage

	EXERCISE	OBJECTIVES	POSITION	ACTIVITY
38	Systematic hold-relaxation and self-massage of the face, neck and head	Relaxation	Sitting	Maintain the position by core activation while doing systematic contraction/relaxation of the various body parts. Self-massage of the face and neck.

Supplementary file 2i: Outdoor sessions

**Content**	**Description**	**Intensity**
	**Introduction (5 minutes):**	
Pre-training balance self-assessment	1) Roll from toe to heel and back again<br>2) Roll from lateral to medial edge of the foot and back again<br>3) "The Kibler", inspired by Kibler, Press & Sciascia, 2006 (1)<br>a) Lift the forefoot and outer board of one foot<br>b) Keep the position while lifting the heal of the floor, weight transferring to the opposite leg<br>c) While standing on one leg, bend the opposite knee and push the heal backwards extending the hip<br>d) Keep the position while bending down on the standing leg (one leg squat)<br>e) Touch the floor with the opposite hand<br>f) Bend the neck<br>e) Slowly roll back to a standing position<br>These assessments can be conducted with eyes open or closed on even or uneven surface.	Low
	**Warm-up (15 minutes):**	
CoreDIST exercises for trunk control in coordination with larger muscle groups.<br><br>**Up to 3x10 repetitions**<br><br>Increase intensity gradually by introducing exercises that demand higher levels of postural control.	**Exercise number 26-28: Squats, squats on one leg and the bear squats**<br>**Up to 3x 10** repetitions<br>Combine the exercise with rolling the spiky ball in your hands in long movements using also the shoulders and middle thorax pointing the fingers to the ground.	Starting with low intensity (below 70% of maximum heart rate or below 10 on Borg Rating of Perceived Exertion (RPE) scale. Gradual increase of pace)<br><br>Gradual increase toward moderate intensity (70% of maximum heart rate or 10-12 on the RPE scale)

Dual tasks may be added in all exercises by singing, counting, rhymes etc.		
	**Exercise number 29-31: The bear calf rise, calf rise and the pole**<br>Combine the exercise with rolling the spiky ball in your hands in long movements using also the shoulders and middle thorax pointing the. fingers down towards the ground.	
	**24) Reach all over in pairs:**<br>This exercise can be conducted alone or in pairs standing back-to-back by:<br>a) Deliver the spiky ball to each other by rotating arms and upper body. Keep your feet and hips still.<br>b) Deliver and catch the ball with extended arms over your head**.**	
	**36) The waiter:**<br>This exercise can be conducted alone, in pairs or while standing in a circle where the ball is delivered to the next person with the arm extended and outwards rotated while keeping the trunk, hip and pelvis in a straight line and weight-shifting towards one leg standing.	
	**37) Pick berries** Pick the spiky ball or pea bag from the ground by taking a step forward or sideways.	
	**35) The bounce:**<br>This exercise can also be done without bouncing the ball (only jumping).	
	**32 og 34) The march**<br>In this exercise the intensity may be increased by running or high knee lifts.<br>**Throwing the ball may simultaneously be conducted (pairwise or in the circle) by:**<br>1) Pushing the ball to each other.<br>2) Throwing the ball side-ways.<br>3) Throwing the ball on the ground.	

	**Main activity, star intervals 32 minutes:**	
4 minutes of high intensity of running, fast walking or exercises at a fast pace and 4 minutes of moderate intensity.<br><br>Repeat up to x4<br><br>Duration, intensity and repetitions should be individualized.	**Running or walking fast** with long strides or fast pace from base to place markers at various distances from the base.<br><br>**Round 1 and 3:** bring a beanbag from base and place at a chosen marker. Count the number of beanbags you were able to please at the various markers.<br><br>**Round 2 and 4:** retrieve the beanbags from the markers and return them to the base.<br><br>Alternatively perform standing exercises using a resistance exercise band and with high intensity: rowing, squats, calf rises, jumping or "picking berries".<br><br>Dual task and cognitive challenges may be added, such as remembering how many red or yellow beanbags you have placed, remembering as many girls' names that start with an A as you can, counting or double your own age for each time you reach a marker.	High intensity (85-95% of maximum heart rate or at least 16 on the RPE scale + moderate intensity.
4 minutes of exercises<br>Up to 3x10 repetitions	Examples:<br>2) Sensory stimulation of hands using the spiky ball<br>3-5) Oculomotor, vestibular exercises and nodding<br>8-9) Exercises for muscle length in the upper extremities (slowly diving and butterfly)<br>20-24) Exercises for selective movement control (hands up, rolling the ball, pelvic walk, the angel, reach all over)	
	**Cool down and re-assessment 8 minutes**	
Post-training balance self-assessment	Retrieve the markers<br>1) Roll from toe to heel<br>2) Roll from lateral to medial edge of the foot	Low intensity

	3) Stand on uneven surface<br>4) Kibler<br><br>These assessments can be conducted with eyes open or closed on even or uneven surface.	

1. Kibler WB, Press J, Sciascia A. The Role of Core Stability in Athletic Function. Sports Medicine. 2006;36(3):189-98.
