# Supplementary material for: A Comprehensive and Structured Follow-Up for Persons With Multiple Sclerosis (CoreDISTparticipation) to Optimize Physical Functions, Health, and Employment: Protocol for a Prospective, Single-Blinded Randomized Controlled Trial and Health Economic Evaluation
Source: JMIR Res Protoc. 2025 Oct 8;14:e74988. doi: 10.2196/74988 (PMC12547332; doi:10.2196/74988)
Supplement: Multimedia Appendix 4 [file resprot_v14i1e74988_app4.pdf]

Supplementary file 4: Conversation guide for meeting between the participant and the NAV-consultant

Introduction to the meeting

This is part of a research project, and this type of meeting is not part of NAV's regular follow-up routines. The goal of this conversation is to provide thorough information about what NAV can assist with now or later, so that you can continue to work, either full-time or part-time. We will write a short summary of this conversation, which will be stored in our system for documentation purposes. This summary will include the topics we discussed and any advice we provided. You can find the summary by logging in to your account at nav.no.

In the following, we will ask you several questions about work and health. It is up to you to decide how much you wish to share with us. It is important that you know that as NAV-consultants we are bound by duty of confidentiality. If, during this conversation, we identify areas where NAV can assist you, we will advise you on where further help can be obtained. Since this is a research project, we will not write a detailed summary of what you tell us. Therefore, if you need further assistance from NAV, you will need to provide the information again. We will revisit this at the end of the conversation. We have allocated 1 hour for this conversation.

Conversation Guide

How would you describe your workday?

- What works well?
- Are there tasks/situations in your workday that are particularly challenging/difficult?
- Possibilities for job accomodations?
  - What accommodations have been made so far, and how have they worked? (Examples: adjustments in tasks, work content, working hours, work pace, organization of work, collaboration with others, alternative tasks, physical layout of the workplace, aids, or other factors)
  - What would be good and supportive help for you in your work?
  - Do you see other tasks/positions at your workplace that might be an option?
- How do you get to/from work?

How is the balance between work and home?

- Do you have energy for daily activities?
- Children?
- Exercise?

What do you find challenging with regards your health condition?

Are there other social factors you need to consider?

- Economy?

- Family?

How would you describe your work environment? Do your colleagues know about your health challenges, and is there a need for them to know?

- In case of absence from work, is your work environment a contributing factor?

Dialogue with employer/manager. Is it easy to talk to your manager about your challenges?

Have you considered whether this is a job you will be able to have in the foreseeable future?

Is there anything else you would like to discuss?

The Way Forward in the Project: Based on what comes up in the conversation – guide further:

- NAV Accommodation and Aids – how to get an appointment here
- NAV Office – how to proceed to get an appointment
- NAV Work Life Center if assistance is needed in dialogue with the employer – how to get help here?
- If other assistance is needed (GP/physio) – ask the participant to contact them themselves

---

After the Meeting: A short note is written, use the following template: Date – A meeting has been conducted with you in connection with the research project "Health, Function, and Work for People with MS". From NAV, nn from NAV Work Life Center and nn from NAV Aids and Accommodation participated. The goal of this conversation is for you as a participant in the research project to receive good information about what NAV can assist with now or later so that you can continue to work, either full-time or part-time. The topics we have discussed are work situation, accommodation possibilities, health situation, social factors, and possible help from NAV. Our further recommendations are:
