# Supplementary material for: A Comprehensive and Structured Follow-Up for Persons With Multiple Sclerosis (CoreDISTparticipation) to Optimize Physical Functions, Health, and Employment: Protocol for a Prospective, Single-Blinded Randomized Controlled Trial and Health Economic Evaluation
Source: JMIR Res Protoc. 2025 Oct 8;14:e74988. doi: 10.2196/74988 (PMC12547332; doi:10.2196/74988)
Supplement: Multimedia Appendix 5 [file resprot_v14i1e74988_app5.pdf]

Nordlandssykehuset
Postboks 1480
8092 Bodø

Deres ref:	Vår ref:	Saksbehandler:	Dato:
	2022/9405		15.12.2022

Tildeling av forskningsmidler fra Helse Nord RHF for 2023 - HNF1687-23

Vi viser til søknad om forskningsmidler til Helse Nord RHF. Prosjektet er tildelt forskningsmidler for 2023 og eventuelt påfølgende år etter oppsettet i tabellen nedenfor, gitt tilfredsstillende fremdrift. Dette gjelder under forutsetning av at alle vilkår oppfylles og at forskningsansvarlig vurderer prosjektet gjennomførbart med foreliggende tildeling. Tildelingen anses som akseptert om ikke annet meldes innen rimelig tid.

Vi ber forskningsansvarlig videreformidle brevet til de involverte, minimum prosjektleder og klinikksjef/instituttleder.

Forskningsansvarlig: Nordlandssykehuset
Klinikk/institutt: Medisinsk klinikk
Prosjektleder: Britt Normann
Tittel: *Tailored follow-up for persons with multiple sclerosis to optimize physical functions, health and employment: a prospective single blinded randomized controlled trial*
Helse Nord prosjektnummer: HNF1687-23
Prosjekttype: Flerårig forskningsprosjekt

År	Totalt tildelings-beløp	Stipend rundsum	Måneder	Stillings-prosent	Utenlands-stipend	Lønn	Drift	Utstyr
**2023**	1 722 075					1 000 000	722 075	
**2024**	1 607 240					1 000 000	607 240	
**2025**	1 559 940					1 240 000	319 940	
**2026**								
**2027**								
**2028**								
**2029**								

Administrativ tilbakemelding på budsjett i søknaden

Sekretariatet gjennomgår søknadens budsjett og foretar nødvendige justeringer i henhold til gjeldende retningslinjer. Sekretariatet har følgende kommentar til ditt budsjett: *Tildelingen er avkortet. Konferansedeltakelse er justert til kr 50 000 i 2023 og 2024 og kr 100 000 i 2025. Det mangler begrunnelse for "egenandel fysioterapi" for reiseutgifter som ikke dekkes av Pasientreiser, og er derfor kuttet, jf. utlysningen side 18.*

Tildeling til drift og utstyr for HF-prosjekt er redusert på grunn av momskompensasjonsordningen, se avsnitt *Nøytral merverdiavgift for helseforetak*.

Aksept av tildeling
Tildelingen til prosjektet er endelig med eventuelle avvik fra omsøkt beløp. Den regnes som akseptert med mindre annet umiddelbart meldes til Helse Nord RHF ved sekretariatet.

Helse Nord RHF forutsetter at forskningsprosjekter gjennomføres i henhold til søknad med de tildelte midler. Dette er viktig av hensyn til progresjon i prosjektet. Dette betyr oppstart slik som skissert i søknaden. Forsinkelser utover en måned etter planlagt oppstart må søkes til sekretariatet. Alle flerårige bevilgninger innvilges gitt tilfredsstillende framdrift.

En forutsetning for tildeling er at nødvendig dokumentasjon innsendes så snart den foreligger.

Der søker får finansiering fra andre eksterne kilder til samme prosjektaktivitet («dobbeltfinansiering»), skal man si fra seg Helse Nord-midler og ta imot forskningsmidler fra ekstern kilde.

Korrespondanse vedrørende prosjektet og tildelingen
Alle henvendelser om tildelingen bes rettet til sekretariatet for Helse Nord RHFs forskningsmidler: forskningsmidler@unn.no. Bruk Helse Nord prosjektnummer som referanse ved henvendelser.

Prosjektleder er ansvarlig for kommunikasjon med sekretariatet. Vi benytter prosjektleders e-postadresse, oppgitt i søknadsskjema, dersom denne endres må det meldes.

Disponering av midlene
Utbetalinger vil skje til forskningsansvarlig, som også er regnskapsansvarlig. Prosjektleder bør planlegge budsjettet med institusjonens økonomiavdeling slik at gjennomførbarheten i prosjektet sikres.

Tildelingen skal brukes til det formål den er bevilget. Tildelt beløp kan avvike fra omsøkt beløp. Lønnsmidler skal benyttes til de personer/stillinger som er spesifisert i søknaden.

Det tildeles en rundsum pr. år for ph.d.-, postdoktor- og forskerstipend. Institusjonen skal gi stipendmottaker den lønn vedkommende har krav på i minimum gitte antall måneder i gitt stillingsprosent, dekke minimum 60 000 kr per årsverk i driftsmidler, samt dekke sosiale utgifter for arbeidsgiver. Institusjonen kan ta eventuelle resterende mellomlegg som dekningsbidrag til HF-et. Universitetene beregner dekningsbidrag etter sine regler. Driftsmidlene forventes å dekke løpende utgifter til pc, programvare, reiser, kurs, konferanser, forskuttering av kostnader til åpen publisering og lignende knyttet til prosjektet. Lønnsmidler som overstiger budsjett kan ikke belastes prosjektet. For spørsmål om avlønning, vises det til Helse Nords søknadsveileder for 2023 pkt. 5.2.

Det tillates ompostering av inntil 10 % av midlene mellom de innvilgede budsjettpostene. Eventuelle ubenyttede midler kan overføres i institusjonens eget regnskap for det enkelte prosjekt fra et år til påfølgende år. For spørsmål angående ovennevnte og prosjektets økonomi generelt / regnskap, kan institusjonens økonomirådgiver(e) kontaktes.

Nøytral merverdiavgift for helseforetak
Innføringen av nøytral merverdiavgift for helseforetak fra 1. januar 2017 berører tildelingen av forskningsmidler. Helseforetak får kompensert merverdiavgift for kjøp av varer og tjenester, derfor er tildelingen justert av Helse Nord RHF. Følgende trekk er foretatt:

- Driftskostnader: 15 %
- Utstyr/investeringer: 20 %

For mer informasjon se Helsedirektoratets nettside.

Endringer i prosjektet
Det skilles mellom to ulike henvendelsesformer til sekretariatet: 1) melding om endring og 2) søknad om endring.

1) Melding om endring
Forsinkelser i prosjektet grunnet lovbestemte permisjoner (som for eksempel sykemeldinger og foreldrepermisjoner) skal meldes, men medfører ingen endringer i utbetalingsplanen. Forsinkelser underveis i prosjektet (utover lovbestemte permisjoner) på inntil 6 måneder, som kun medfører endringer i aktivitetsplanens tidsangivelse, skal meldes i årsrapporten.

Forskningsansvarlig skal forlenge prosjektperioden automatisk for endringer som kun medfører krav om melding.

2) Søknad om endring
Ved større endringer skal det sendes endringssøknad til Helse Nord RHF ved sekretariatet. Dette gjelder for eksempel utsatt/forsinket oppstart av prosjektet, endring av prosjektleder, bytte av navngitt kandidat, tilsetting av ny kandidat (der første kandidat har sagt opp sin stilling), endring i stillingsandel, permisjoner (ikke lovbestemte), endring i aktivitetsplan (dvs. alt som påvirker prosjektet innholdsmessig), langvarige forsinkelser underveis i prosjektet (ut over 6 måneder) selv om innholdet i aktivitetsplanen ikke endres (merk gjelder ikke lovbestemte permisjoner, jf. punkt 1).

Innvilget endringssøknad må foreligge før prosjektet kan videreføres/fullføres.

Innsending av dokumentasjon
Endelig godkjenning fra REK og Statens legemiddelverk skal ettersendes så snart den foreligger (gjelder alle prosjekter som omfattes av helseforskningsloven og legemiddelloven).

Ph.d.-stipendiater må dokumentere opptak ved UiT eller Nord universitet innen tre måneder etter tiltredelse. Ph.d.-kontrakt må ettersendes sekretariatet snarest.

Tildeling av postdoktorstipend er gjort under forutsetning av innlevering av ph.d.-avhandling senest 31.12.2022, og avlagt ph.d.-grad innen 1.9.2023.

Ved manglende dokumentasjon av nødvendige godkjenninger eller opptak til ph.d.-program, vil utbetalingen stoppes.

Søknader uten navngitt kandidat
Krav til stipendiat som er angitt i søknadsveilederen for 2023 gjelder også ved ansettelse i prosjekt uten navngitt kandidat. Dette gjelder for eksempel krav til kompetanse, avlagt master-/ph.d.-grad og krav tilknyttet tilsetting i postdoktorstipend.

Så snart navn og starttidspunkt er klart skal dette meldes til sekretariatet med kopi av kandidatens CV og vitnemål.

Ansettelse og arbeidsplass
Forskningsansvarlig har arbeidsgiveransvaret for forsker/stipendiat og det organisatoriske ansvaret for gjennomføringen av prosjektet.

Ved tildeling av stipend skal det inngås avtale mellom klinikk/avdeling og stipendiat/forsker om avsatt arbeidstid til forskning. Det er laget en mal for slik avtale som er innført i helseforetak i Helse Nord: *Avtale ved tilsetting i forskningsprosjekt finansiert av Helse Nord.* Denne finnes i Docmap, dok.nr. AV0703. For institusjoner utenfor Helse Nord RHF, kan avtalemalen lastes ned fra Helse Nord RHF sine nettsider.

Utøvende forsker, særlig ph.d.- og postdoktorstipendiat, skal fysisk ha arbeidsplass ved forskningsansvarliges lokaler. Dette for å være del av og bygge forskningsmiljø. Unntaksvis kan dette fravikes, primært innen regionen, og da etter *særskilt* søknad. Det gis ikke midler til stipendiat/forsker som er tenkt å være utenfor regionen.

Eierskap til utstyr
Utstyr av varig verdi som kjøpes inn for prosjektmidler, eies av forskningsansvarlig. Denne påtar seg også ansvaret for videre drift og vedlikehold av utstyret i dets normale levetid.

Forfatteradresser og publisering
Ved publisering gjelder følgende regler for forfatteradresser, jf. vedtak i Nasjonal samarbeidsgruppe for helseforskning (NSG), 02.11.2011:

1. En institusjon skal oppgis som adresse i en publikasjon dersom den har gitt et nødvendig og vesentlig bidrag til eller grunnlag for en forfatters medvirkning til det publiserte arbeidet.
2. Samme forfatter skal oppgi også andre institusjoners adresser dersom disse i hvert enkelt tilfelle også tilfredsstiller kravet i punkt 1.
3. Et ansettelsesforhold eller et veiledningskrav kan regnes som grunnlag for kreditering av en institusjon, *hvis kravet i punkt 1 er innfridd*.
4. Universitets - eller høgskoletilsatt i kombinert vitenskapelig stilling med helseforetak/sykehus skal som hovedregel oppgi adresse både til egen akademisk institusjon (universitet eller høgskole) og helseforetaket. Dette gjelder uavhengig av stillingsandel ved universitet/ høyskole og finansiering av stillingen.

Finansiering av stilling/prosjekt regnes som et nødvendig og vesentlig bidrag til og grunnlag for prosjektleders og eventuelle stipendmottakeres medvirkning til den publiserte artikkelen. Dette innebærer at alle vitenskapelige publikasjoner fra prosjektet skal ha forfatteradresser i et helseforetak i Helse Nord eller i Helse Nord RHF. Dette gjelder **prosjektleder og alle andre forskere med finansiering fra prosjektet**. Personer som ikke har formell tilsetting ved et helseforetak i Helse Nord skal fortrinnsvis bruke samarbeidende klinikk som forfatteradresse, eventuelt sammen med andre adresser (dobbel adresse). Alternativt kan Forsknings- og utdanningssenter, UNN HF, eller Helse Nord RHF benyttes. Dersom det er spørsmål til publisering og adressering, ta kontakt med sekretariatet.

Åpen publisering
Vitenskapelige artikler skal publiseres i poenggivende fagfellevurderte tidsskrifter, jf. Register over vitenskapelige publiseringskanaler.

Helse Nord RHF krever at vitenskapelige artikler publiseres åpent tilgjengelig. Primært skal åpne tidsskrifter/plattformer benyttes. Helse Nord støtter ikke hybridtidsskrifter. Det kan ikke brukes forskningsmidler til å betale for å publisere artikler åpent i tidsskrifter der artikkelen ellers ville vært bak betalingsmur for leseren.[39] Dersom det publiseres i tradisjonelle abonnementsfinansierte tidsskrifter, skal det fagfellevurderte manuset egenarkiveres uten forsinkelse.

Ved publisering skal artikkelen registreres i Cristin og siste fagfellevurderte versjon av manuset lastes opp der.

Vancouver-reglene for forfatterskap skal følges. Se for eksempel forskningsetikk.no

Helse Nord har et fond for å dekke forfatternes kostnader ved åpen publisering, etter gitte regler (https://helse-nord.no/forskning-og-innovasjon).

Rapportering
Fremdriften skal rapporteres årlig. Prosjektleder er ansvarlig for den faglige rapporteringen. Denne leveres gjennom eRapport med frist medio januar (gjennom tilsendt lenke). Rapporter publiseres på nett på http://forskningsprosjekter.ihelse.net. Forskningsansvarlig rapporterer samlet for økonomien i alle tildelte prosjekter.

Etter prosjektets slutt, skal det leveres en sluttrapport. Som hovedregel leveres sluttrapport når prosjektets finansieringsperiode er over. Unntaksvis kan prosjektleder velge årsrapport dersom prosjektet ikke er ferdigstilt. Ved sluttrapport skal også regnskapet avsluttes og eventuelle ubrukte midler returneres til Helse Nord RHF. Sekretariatet kan også legge inn tvungen sluttrapportering for et prosjekt.

For ph.d.-prosjekt ønsker Helse Nord RHF å få tilsendt den godkjente avhandlingen når den foreligger. Avhandlingen sendes: Helse Nord RHF v/forskningsdirektør, 8038 Bodø.

[39] De institusjonelle overgangsavtalene om åpen publisering (les&publiser-avtalene) kan benyttes.

Vilkår for tildelingen
Se for øvrig utlysningen for 2023 for utfyllende informasjon og oversikt over fullstendige vilkår som gjelder for tildelingen.

Vi minner om at godkjenninger og registreringer må være i orden før igangsetting av prosjektet:

- REK-godkjenning (gjelder alle prosjekter som omfattes av helseforskningsloven)
- Personvernombudet
- Helsedirektoratet (ved klinisk utprøving av medisinsk utstyr og genforskningsprosjekter som omfattes av bioteknologiloven)
- Statens legemiddelverk (ved legemiddelutprøving)
- Relevante register (ClinicalTrials.gov, EudraCT, Helsenorge.no, ev. institusjonsinterne systemer)

For prosjekter forankret ved helseforetak minner vi om muligheten for regional forskningsstøtte ved Klinisk forskningsavdeling (KFA), UNN. Avdelingen tilbyr blant annet veiledning i forbindelse med planlegging og gjennomføring av prosjekt, datafangstløsninger, statistikkhjelp og monitorering av kliniske studier. Det tilbys også flere forskerkurs.

Vi ønsker lykke til med forskningsprosjektet.

Vennlig hilsen

Sekretariatet for Helse Nord RHFs forskningsmidler
forskningsmidler@unn.no

Dokumentet er elektronisk godkjent og kan derfor være uten signatur.

Vedlegg:
Karakterer og skriftlig kommentar
Informasjon om søknadsmassen – Forskningsmidler for 2023

Translation of Letter confirming allocation of funds dated 15.12.2022

Allocation of Research Funds from Helse Nord RHF for 2023 - HNF1687-23

We refer to the application for research funds to Helse Nord RHF. The project has been allocated research funds for 2023 and possibly subsequent years according to the setup in the table below, given satisfactory progress. This is conditional on all terms being met and the research manager assessing the project as feasible with the current allocation. The allocation is considered accepted unless otherwise notified within a reasonable time.

We ask the research manager to forward the letter to the involved parties, at a minimum the project manager and the clinic/department head.

Research Manager: Nordland Hospital
Clinic/Department: Medical Clinic
Project Manager: Britt Normann
Title: Tailored follow-up for persons with multiple sclerosis to optimize physical functions, health, and employment: a prospective single-blinded randomized controlled trial
Helse Nord Project Number: HNF1687-23
Project Type: Multi-year research project

Year	Total Allocation Amount (NOK)	Scholar-ship Lump Sum	Months	Percentage of Position	Foreign Scholarship	Salary (NOK)	Operations (NOK)	Equip ment
**2023**	1 722 075					1 000 000	722 075	
**2024**	1 607 240					1 000 000	607 240	
**2025**	1 559 940					1 240 000	559 940	

Administrative Feedback on Budget in the Application

The secretariat reviews the application’s budget and makes necessary adjustments according to current guidelines. The secretariat has the following comment on your budget: The allocation has been reduced. Conference participation has been adjusted to NOK 50,000 in 2023 and 2024, and NOK 100,000 in 2025. There is no justification for the “co-payment for physiotherapy” for travel expenses not covered by Patient Travel, and it has therefore been cut, cf. the announcement on page 18.

Allocation for Operations and Equipment for the HF Project has been Reduced Due to the VAT Compensation Scheme. See the section on Neutral VAT for Health Enterprises.

Acceptance of Allocation

The allocation to the project is final with any changes from the applied amount. It is considered accepted unless otherwise immediately notified to Helse Nord RHF by the secretariat. Helse Nord RHF assumes that research projects are carried out according to the application with the allocated funds. This is important for the progress of the project. This means starting as outlined in the application. Delays beyond one month after the planned start must be applied for to the secretariat. All multi-year grants are awarded given satisfactory progress.

A condition for the allocation is that the necessary documentation is submitted as soon as it is available. If the applicant receives funding from other external sources for the same project activity ("double funding"), they must relinquish Helse Nord funds and accept research funds from the external source.

Correspondence Regarding the Project and Allocation

All inquiries about the allocation should be directed to the secretariat for Helse Nord RHF's research funds: forskningsmidler@unn.no. Use the Helse Nord project number as a reference in inquiries. The project manager is responsible for communication with the secretariat. We use the project manager's email address provided in the application form; if this changes, it must be reported.

Allocation of Funds

Payments will be made to the research manager, who is also responsible for accounting. The project manager should plan the budget with the institution's finance department to ensure the feasibility of the project.

The allocation must be used for the purpose for which it was granted. The allocated amount may differ from the applied amount. Salary funds must be used for the persons/positions specified in the application. A lump sum per year is allocated for Ph.D., postdoctoral, and research scholarships. The institution must provide the scholarship recipient with the salary they are entitled to for a minimum number of months at a given percentage of the position, cover a minimum of NOK 60,000 per full-time equivalent in operating funds, and cover social expenses for the employer. The institution can take any remaining balance as a contribution margin to the HF. Universities calculate the contribution margin according to their rules.

Operating funds are expected to cover ongoing expenses for PCs, software, travel, courses, conferences, advance costs for open publishing, and similar project-related expenses. Salary funds exceeding the budget cannot be charged to the project. For

questions about remuneration, refer to Helse Nord's application guide for 2023, section 5.2.

Reallocation of up to 10% of the funds between the approved budget items is allowed. Any unused funds can be transferred to the institution's own accounts for the individual project from one year to the next. For questions regarding the above and the project's finances in general/accounting, the institution's financial advisor(s) can be contacted.

Neutral VAT for Health Enterprises

The introduction of neutral VAT for health enterprises from January 1, 2017, affects the allocation of research funds. Health enterprises are compensated for VAT on purchases of goods and services; therefore the allocation has been adjusted by Helse Nord RHF. The following deductions have been made:

- Operating costs: 15%
- Equipment/investments: 20%

For more information, see the Directorate of Health's website.

Changes in the Project

There are two different forms of communication with the secretariat: 1) notification of change and 2) application for change.

1. **Notification of Change** Delays in the project due to statutory leave (such as sick leave and parental leave) must be reported but do not result in changes to the payment plan. Delays during the project (beyond statutory leave) of up to 6 months, which only result in changes to the activity plan's timeline, must be reported in the annual report. The research manager should automatically extend the project period for changes that only require notification.
2. **Application for Change** For major changes, a change application must be submitted to Helse Nord RHF by the secretariat. This applies, for example, to postponed/delayed project start, change of project manager, replacement of a named candidate, hiring a new candidate (if the first candidate has resigned), change in percentage of position, non-statutory leave, changes in the activity plan (i.e., anything that affects the project's content), long-term delays during the project (beyond 6 months) even if the content of the activity plan does not change (note this does not apply to statutory leave, see point 1).

An approved change application must be in place before the project can continue/be completed.

Submission of Documentation

Final approval from REK and the Norwegian Medicines Agency must be submitted as soon as it is available (applies to all projects covered by the Health Research Act and the Medicines Act). Ph.D. candidates must document admission to UiT or Nord University within three months after starting. The Ph.D. contract must be submitted to the secretariat as soon as possible.

The allocation of postdoctoral scholarships is conditional on the submission of the Ph.D. thesis by 31.12.2022, and the Ph.D. degree being awarded by 1.9.2023.

If the necessary approvals or admission to the Ph.D. program are not documented, the payment will be stopped.

Applications Without a Named Candidate

The requirements for the candidate specified in the application guide for 2023 also apply when hiring for a project without a named candidate. This includes requirements for competence, a completed master's/Ph.D. degree, and requirements related to the appointment of a postdoctoral scholarship.

As soon as the name and start date are known, this must be reported to the secretariat with a copy of the candidate's CV and diploma.

Employment and Workplace

The research manager has the employer responsibility for the researcher/candidate and the organizational responsibility for the implementation of the project.

When awarding a scholarship, an agreement must be made between the clinic/department and the candidate/researcher regarding the allocated working time for research. A template for such an agreement has been introduced in health enterprises in Helse Nord: Agreement for Employment in Research Projects Funded by Helse Nord. This can be found in Docmap, document no. AV0703. For institutions outside Helse Nord RHF, the agreement template can be downloaded from Helse Nord RHF's website.

The performing researcher, especially Ph.D. and postdoctoral candidates, must physically have a workplace at the research manager's premises. This is to be part of and build the research environment. Exceptions can be made, primarily within the region, and then by special application. No funds are provided for candidates/researchers intended to be outside the region.

Ownership of Equipment

Equipment of lasting value purchased with project funds is owned by the research manager. They also take responsibility for the continued operation and maintenance of the equipment during its normal lifespan.

Author Addresses and Publishing

When publishing, the following rules for author addresses apply, according to the decision of the National Cooperation Group for Health Research (NSG), 02.11.2011:

1. An institution should be listed as an address in a publication if it has made a necessary and significant contribution to or basis for an author's participation in the published work.
2. The same author should also list other institutions' addresses if they also meet the requirement in point 1 in each case.
3. An employment relationship or a supervision requirement can be considered a basis for crediting an institution if the requirement in point 1 is met.
4. University or college employees in a combined scientific position with a health enterprise/hospital should generally list both their academic institution (university or college) and the health enterprise as addresses. This applies regardless of the percentage of the position at the university/college and the funding of the position.

Funding of Position/Project

Funding of the position/project is considered a necessary and significant contribution to and basis for the project manager's and any scholarship recipients' participation in the published article. This means that all scientific publications from the project must have author addresses in a health enterprise in Helse Nord or in Helse Nord RHF. This applies to the project manager and all other researchers funded by the project. Persons who do not have a formal appointment at a health enterprise in Helse Nord should preferably use the collaborating clinic as the author address, possibly together with other addresses (double address). Alternatively, the Research and Education Center, UNN HF, or Helse Nord RHF can be used. If there are questions about publishing and addressing, contact the secretariat.

Open Access Publishing

Scientific articles must be published in peer-reviewed journals that award points, cf. Register of Scientific Publishing Channels. Helse Nord RHF requires that scientific

articles be published openly accessible. Primarily, open journals/platforms should be used. Helse Nord does not support hybrid journals. Research funds cannot be used to pay for open access publishing in journals where the article would otherwise be behind a paywall for the reader. If published in traditional subscription-funded journals, the peer-reviewed manuscript must be self-archived without delay.

When publishing, the article must be registered in Cristin, and the latest peer-reviewed version of the manuscript must be uploaded there. The Vancouver rules for authorship must be followed. See, for example, forskningsetikk.no. Helse Nord has a fund to cover authors' costs for open access publishing, according to given rules (https://helse-nord.no/forskning-og-innovasjon).

Reporting

Progress must be reported annually. The project manager is responsible for the professional reporting. This is delivered through eRapport with a deadline in mid-January (via a sent link). Reports are published online at http://forskningsprosjekter.ihelse.net. The research manager reports collectively on the finances of all allocated projects.

After the project's end, a final report must be submitted. As a rule, the final report is submitted when the project's funding period is over. Exceptionally, the project manager can choose an annual report if the project is not completed. With the final report, the accounts must also be closed, and any unused funds returned to Helse Nord RHF. The secretariat can also impose mandatory final reporting for a project.

For Ph.D. projects, Helse Nord RHF wishes to receive the approved thesis when it is available. The thesis is sent to: Helse Nord RHF v/forskningsdirektør, 8038 Bodø. The institutional transition agreements on open access publishing (read & publish agreements) can be used.

Terms of the Allocation

Please refer to the 2023 announcement for detailed information and a complete overview of the terms applicable to the allocation.

We remind you that approvals and registrations must be in order before starting the project:

- REK approval (applies to all projects covered by the Health Research Act)
- Data Protection Officer
- Directorate of Health (for clinical trials of medical devices and genetic research projects covered by the Biotechnology Act)
- Norwegian Medicines Agency (for drug trials)

- Relevant registers (ClinicalTrials.gov, EudraCT, Helsenorge.no, or internal institutional systems)

For projects anchored at health enterprises, we remind you of the possibility of regional research support at the Clinical Research Department (KFA), UNN. The department offers guidance in planning and implementing projects, data capture solutions, statistical assistance, and monitoring of clinical studies. Several research courses are also offered.

We wish you the best of luck with your research project.

Best regards,
The Secretariat for Helse Nord RHF's Research Funds
forskningsmidler@unn.no

This document is electronically approved and may therefore be without a signature.

Attachments:

Grades and Brief Written Comment

Information on the Application Pool -Research funds for 2023

Karakterer og kort skriftlig kommentar

Den skriftlige kommentaren er ikke ment som begrunnelse for karaktersettingen, men som kommentarer og forslag med tanke på prosjekt- og søknadsforbedringer.

Forskningsansvarlig: Nordlandssykehuset
Klinikk/institutt: Medisinsk klinikk
Prosjektleder: Britt Normann
Tittel: *Tailored follow-up for persons with multiple sclerosis to optimize physical functions, health and employment: a prospective single blinded randomized controlled trial*
Helse Nord prosjektnummer: HNF1687-23
Prosjekttype: Flerårig forskningsprosjekt

Vurderingskomiteen har gitt følgende skriftlige kommentar til søknaden:

God søknad som også inkluderer risikovurderinger. Det er en meget kompleks intervensjon, som til slutt følger e-støttet egentrening. Det er viktig at det forskes på denne type intervensjoner, og det er positivt at det planlegges helseøkonomisk evaluering. Valg av utfallsmål er noe svakt begrunnet, det stilles spesielt spørsmål om valget av endepunkter er optimalt. Komiteen savner mer beskrivelse av «usual care» for pasientgruppen, inkludert hvilke intervensjoner som gis som «usual care» til pasientene i de aktuelle kommunene. Det savnes også vurderinger rundt påvirkning av utfallsmål og mulighet for bias da denne pasientgruppen undersøkes relativt hyppig. Relativt sterk faglig bakgrunn for søker.

Søknaden er gitt følgende karakterer:

Kvalitet:	3,7
Nytte:	3,6
Samlet karakter:	3,65

Din søknad er vurdert av komité 2. Samlet karakter til den sist innvilgede søknaden: 3,55 (komité 1), 3,45 (komité 2) og 3,65 (komité 3).

Vi viser til vedlegg om *informasjon om søknadsmassen* for å se hvordan søknaden er vurdert i forhold til andre søknader.

Translation of Peer review report

Grades and Brief Written Comment

The written comment is not intended as a justification for the grading but as comments and suggestions for project and application improvements.

Research Manager: Nordland Hospital
Clinic/Department: Medical Clinic
Project Manager: Britt Normann
Title: Tailored follow-up for persons with multiple sclerosis to optimize physical functions, health, and employment: a prospective single-blinded randomized controlled trial
Helse Nord Project Number: HNF1687-23
Project Type: Multi-year research project

The evaluation committee has provided the following written comment on the application:
Good application that also includes risk assessments. It is a very complex intervention, which ultimately follows e-supported self-training. It is important to research this type of intervention, and it is positive that a health economic evaluation is planned. The choice of outcome measures is somewhat weakly justified, and there are specific questions about whether the choice of endpoints is optimal. The committee misses more description of “usual care” for the patient group, including which interventions are given as “usual care” to patients in the relevant municipalities. There are also missing assessments regarding the impact of outcome measures and the possibility of bias as this patient group is relatively frequently examined. The applicant has a relatively strong professional background.

The application has been given the following grades:
Quality: 3.7
Benefit: 3.6
Overall Grade: 3.65

Your application was evaluated by Committee 2. The overall grade for the last approved application:
3.55 (Committee 1), 3.45 (Committee 2), and 3.65 (Committee 3).

Please refer to the attachment for information on the application pool to see how your application was assessed in relation to other applications

Informasjon om søknadsmassen

Innkomne søknader for 2023

Innen søknadsfristen 1. september 2022 var det kommet inn 89 søknader. Søknadene er behandlet slik:

- Åtte søknader ble avvist på grunn av formelle feil. To klaget på vedtaket, avvisningen ble opprettholdt.
- Elleve søknader er behandlet i komité for start- og fullføringsstipend. Fem søknader om startstipend og seks om fullføringsstipend ph.d.-grad.
- En søknad (utenlandsstipend) er vurdert i hovedkomiteen.
- Totalt 69 søknader er vurdert i vurderingskomiteene: komité 1 (24), komité 2 (23) og komité 3 (22).

Tildelingsutvalget i Helse Nord hadde møte 29. november 2022 og fattet vedtak om tildeling av forskningsmidler for 2023. Totalt 44 nye prosjekter ble tildelt midler. En oversikt over tildelingene for 2023 ble publisert på Helse Nords nettsider 2. desember 2022.

Det er utarbeidet histogram for kvalitet og nytte i henholdsvis komité 1, 2 og 3:

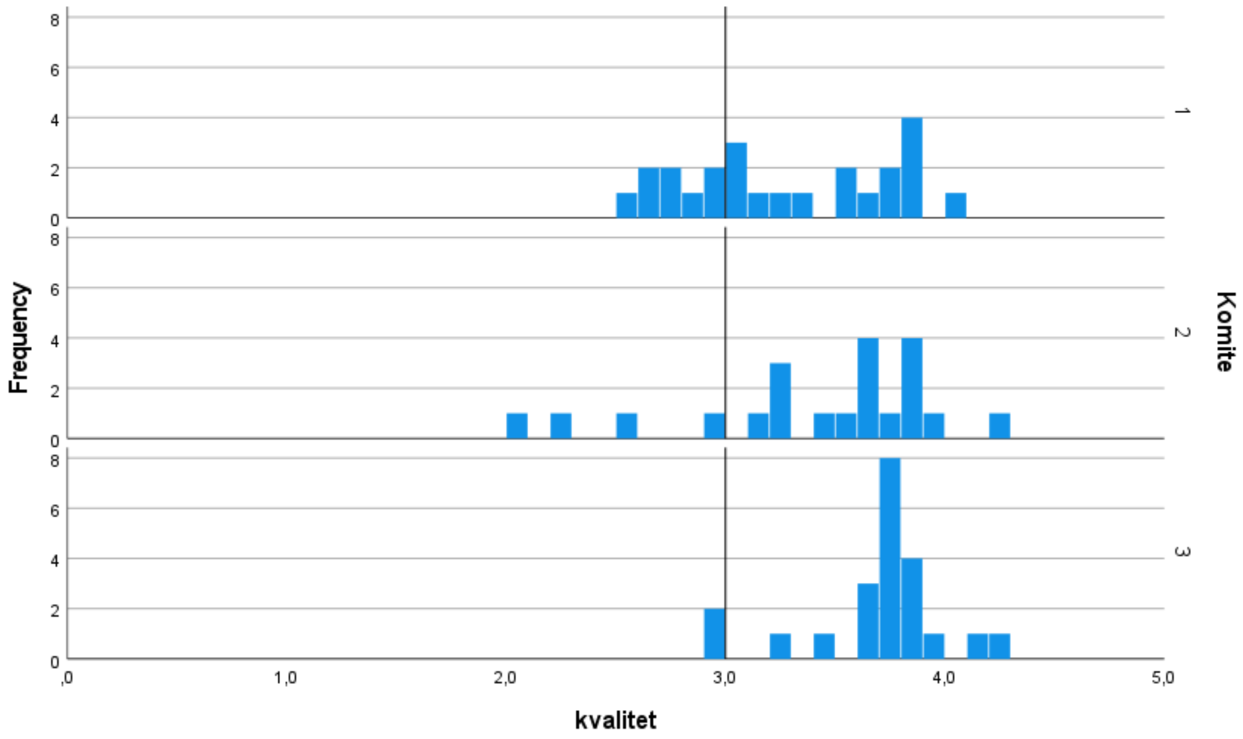

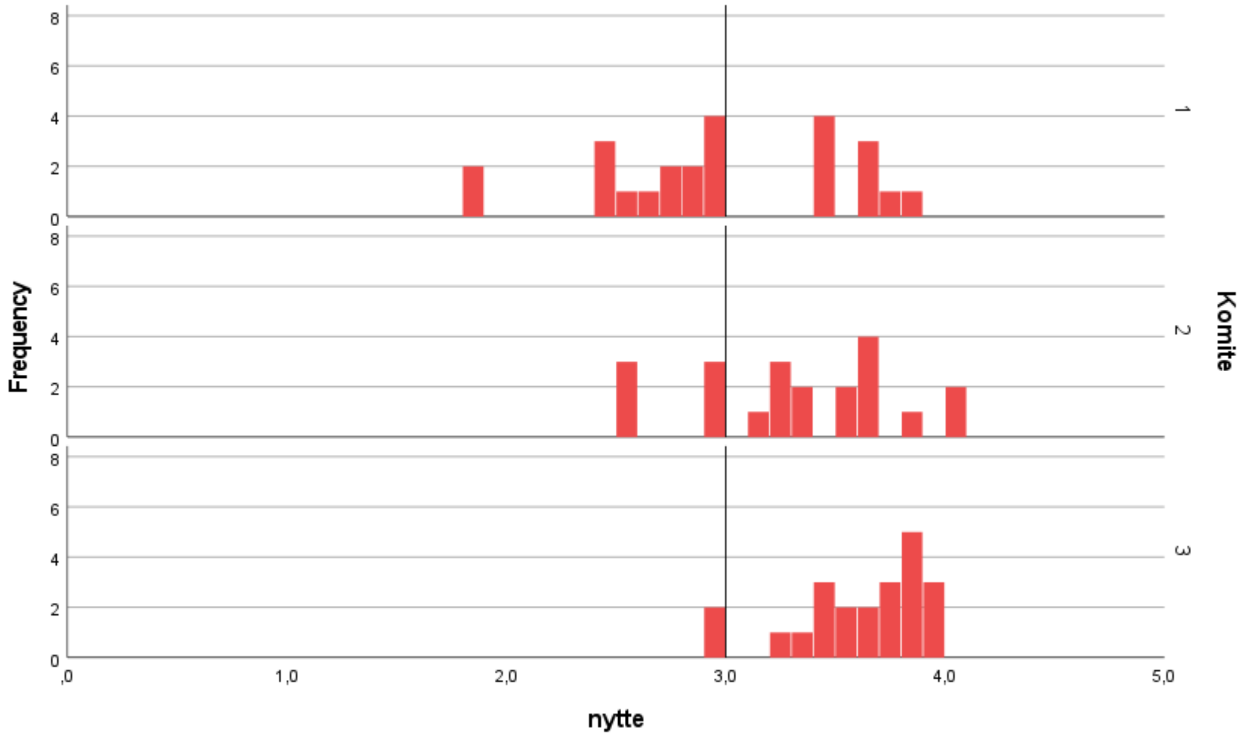

I tabellen nedenfor vises fordelingen av karakterene etter persentiler som viser hvor mange av karakterene som er lavere/høyere enn grensene:

	Komité 1		Komité 2		Komité 3	
	Kvalitet	Nytte	Kvalitet	Nytte	Kvalitet	Nytte
5 %	2,5	1,8	2,0	2,5	2,9	2,9
10 %	2,6	2,1	2,3	2,5	3,0	3,0
25 %	2,8	2,5	3,2	2,9	3,6	3,4
50 %	3,2	2,9	3,6	3,3	3,7	3,7
75 %	3,7	3,4	3,8	3,6	3,8	3,8
90 %	3,8	3,7	3,9	4,0	4,0	3,9
95 %	4,0	3,8	4,2	4,0	4,2	3,9

Samlet karakter til den sist innvilgede søknaden: 3,55 (komité 1), 3,45 (komité 2) og 3,65 (komité 3).

Translation of Information on the Application Pool

Applications Received for 2023

By the application deadline of September 1, 2022, 89 applications had been received. The applications were processed as follows:

- Eight applications were rejected due to formal errors. Two appealed the decision, but the rejection was upheld.
- Eleven applications were processed in the committee for start and completion scholarships. Five applications for start scholarships and six for completion scholarships for Ph.D. degrees.
- One application (foreign scholarship) was assessed by the main committee.
- A total of 69 applications were evaluated by the evaluation committees: Committee 1 (24), Committee 2 (23), and Committee 3 (22).

The allocation committee in Helse Nord met on November 29, 2022, and made decisions on the allocation of research funds for 2023. A total of 44 new projects were awarded funds. An overview of the allocations for 2023 was published on Helse Nord's website on December 2, 2022.

Histograms for quality and benefit have been prepared for Committees 1, 2, and 3, respectively:

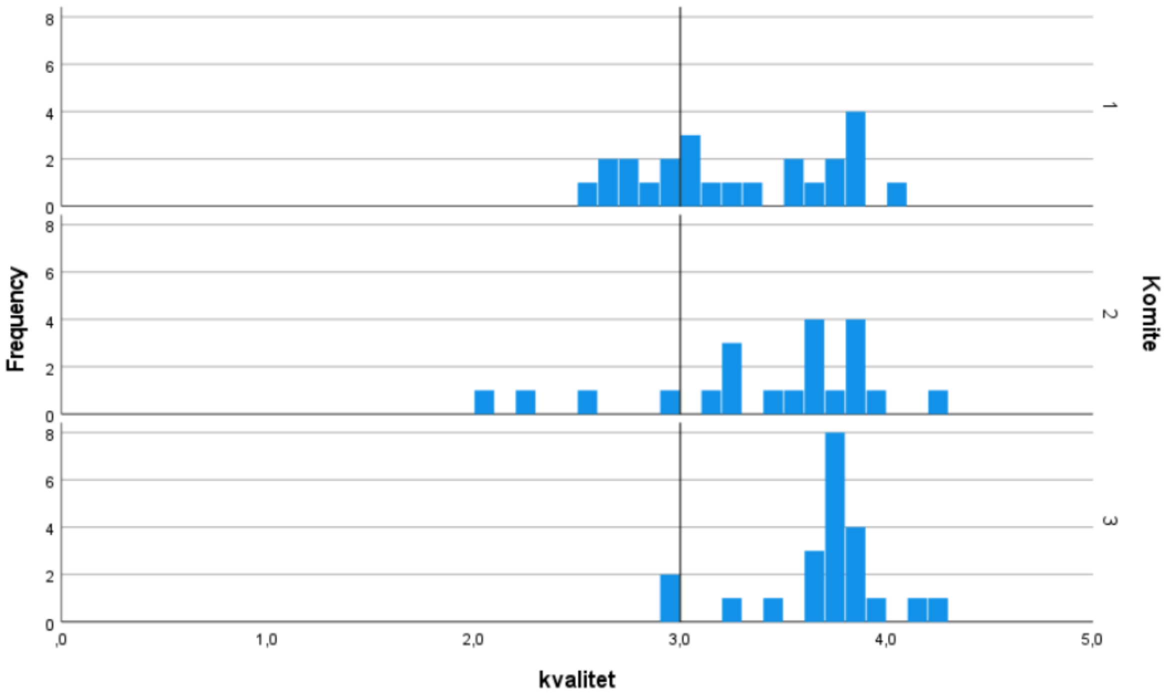

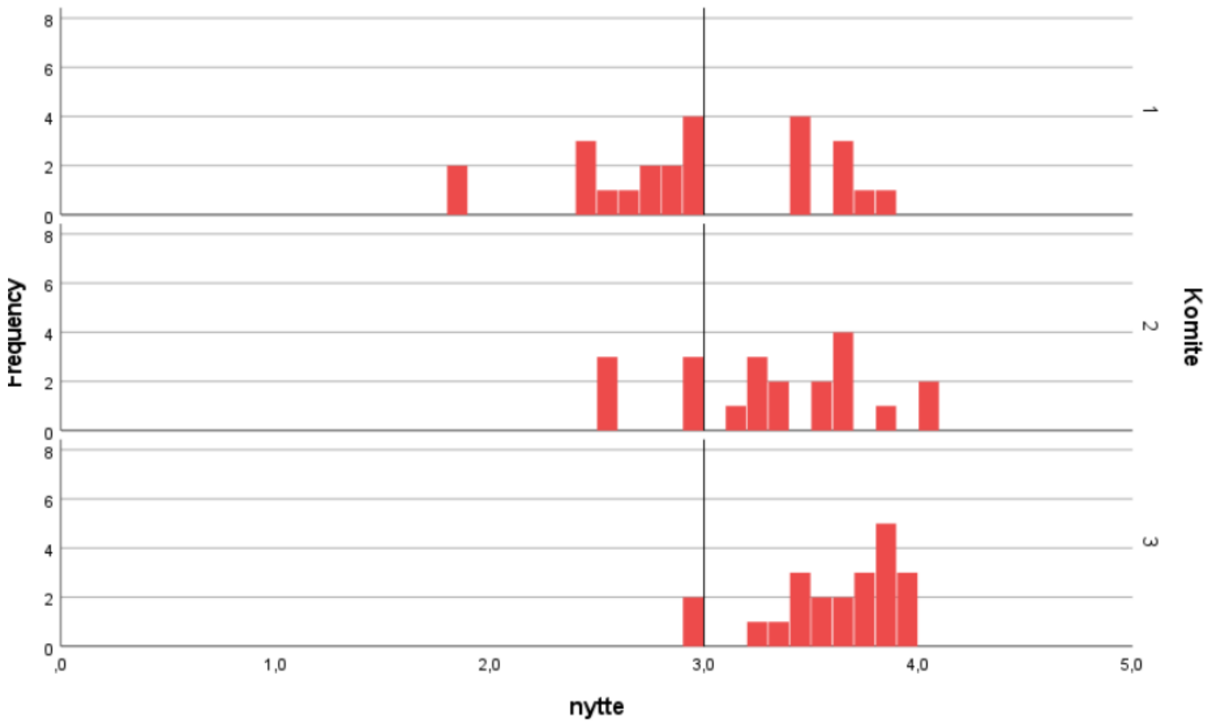

In the table below, the distribution of grades by percentiles is shown, indicating how many of the grades are lower/higher than the thresholds. Kvalitet=Quality, Nytte=Benefit

	Komité 1		Komité 2		Komité 3	
	Kvalitet	Nytte	Kvalitet	Nytte	Kvalitet	Nytte
5 %	2,5	1,8	2,0	2,5	2,9	2,9
10 %	2,6	2,1	2,3	2,5	3,0	3,0
25 %	2,8	2,5	3,2	2,9	3,6	3,4
50 %	3,2	2,9	3,6	3,3	3,7	3,7
75 %	3,7	3,4	3,8	3,6	3,8	3,8
90 %	3,8	3,7	3,9	4,0	4,0	3,9
95 %	4,0	3,8	4,2	4,0	4,2	3,9

Overall score for the most recently approved application: 3.55 (committee 1), 3.45 (committee 2), and 3.65 (committee 3).
